# Microstructure of amide-functionalized polyethylenes determined by NMR relaxometry

**DOI:** 10.1039/d5sc08878j

**Published:** 2026-01-29

**Authors:** Shira Haber, Nicodemo R. Ciccia, Zhengxing Peng, Feipeng Yang, Julia Im, Mutian Hua, Sophia N. Fricke, Raynald Giovine, Brett A. Helms, Cheng Wang, John F. Hartwig, Jeffrey A. Reimer

**Affiliations:** a Materials Sciences Division, Lawrence Berkeley National Laboratory 1 Cyclotron Road Berkeley CA 94720 USA shira.haber@biu.ac.il reimer@berkeley.edu; b Department of Chemistry, University of California, Berkeley Berkeley CA 94720 USA jhartwig@berkeley.edu; c Division of Chemical Sciences, Lawrence Berkeley National Laboratory Berkeley CA 94720 USA; d Advanced Light Source, Lawrence Berkeley National Laboratory 1 Cyclotron Road Berkeley CA 94720 USA; e National Synchrotron Light Source II, Brookhaven National Laboratories Upton NY 11973 USA; f Department of Chemical and Biomolecular Engineering, University of California Berkeley Berkeley CA 94720 USA; g Pines Magnetic Resonance Center, Core NMR Facility, College of Chemistry, University of California, Berkeley Berkeley CA 94720 USA; h The Molecular Foundry, Lawrence Berkeley National Laboratory Berkeley CA 94720 USA

## Abstract

Amidation of polyethylenes creates a range of amide-containing materials with enhanced properties, but the effect of these functional groups on the microstructure of these new materials is not known. Here we employ solid-state nuclear magnetic resonance (NMR) techniques to analyze the microstructure of amide-modified polyethylenes. While a decrease in crystallinity was observed with increasing amounts of functionalization, we found by measuring the chain mobility of the crystalline, amorphous, and interphasial regions of the polyethylenes with NMR relaxation techniques that the grafted amidyl groups partition into the rigid amorphous fraction (RAF) between the crystalline and amorphous regions. The chemical specificity of these NMR experiments creates precise assessments of the location of functional groups within the materials. Together, these insights into the microstructure and morphology of amide-containing polyethylenes lay a foundation for a deeper understanding of the structure and properties of functional polyolefins.

## Abstract 2

NMR relaxation studies reveal that pendant amidyl groups on polyethylenes alter microstructure and chain dynamics. These groups are localized in the rigid amorphous fraction of the polymers and lead to changes in bulk properties.

## Graphical abstract

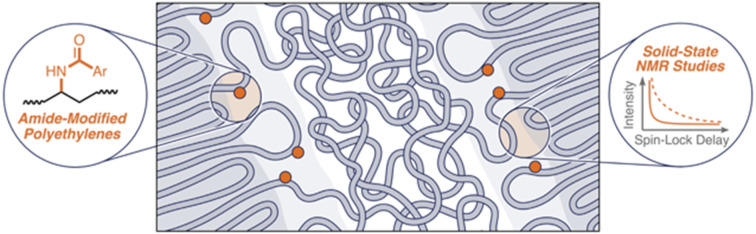

## Introduction

Functional polyethylenes contain low percentages of polar groups along the hydrocarbon backbone that impart favorable bulk and surface properties, and these materials are used in a range of everyday applications.^1^ The structure and composition of these polymers, including the identity and distribution of the functional groups and the architecture of the polymer, influence materials properties; altering these factors enables the synthesis of bespoke materials for specific applications. Progress has been made toward the development of diverse functional polyethylenes by polymerization, copolymerization, and post-polymerization modification to create materials possessing a range of properties.^2–5^ Given the value of these properties, a detailed understanding of the effect of low percentages of functional groups on the microstructure and crystallinity of these polymers is crucial to inform the rational design of new, useful materials.

Extensive studies have shown that small functional groups on polyethylene, such as alcohols or ketones, become partially incorporated into the crystallites without large impacts on overall crystallinity, whereas large pendant groups, such as esters, are excluded from the crystallites and reduce crystallinity.^6–24^ Specifically, the presence of bulky groups on the polymer backbone results in shortened segments along the chain that are able to align in the crystalline domain, thus reducing the size of the crystallites.^21–24^ Other work has shown that the interphasial domain (also referred to as the rigid amorphous fraction (RAF)) between the crystalline and amorphous domains behaves as a distinct phase with varying thicknesses that result from polymer structure and crystallization conditions.^20,22,25–38^ While Raman and differential scanning calorimetry (DSC) techniques have shown that short-chain aliphatic branches in polyethylenes often reside in the RAF,^25,28^ less is known about the partitioning of polar groups between the RAF and amorphous domain in functional polyethylenes and how this partitioning impacts bulk properties.^11,25^

Solid-state nuclear magnetic resonance (NMR) relaxometry techniques, specifically spin-lattice relaxation (*T*_1_) and spin-lattice relaxation in the rotating frame (*T*_1ρ_) measurements, enable analysis of each polymer phase by measuring chain mobility; chains in the crystalline region relax slowly, whereas chains in the amorphous region relax rapidly, and chains in the interphase region relax with intermediate rates.^32–40^ Importantly, the chemical specificity of NMR measurements makes possible distinct analysis of polymer chains and functional groups, and, thus, could provide insights into the colocalization of these groups and different phases.

**Fig. 1:**
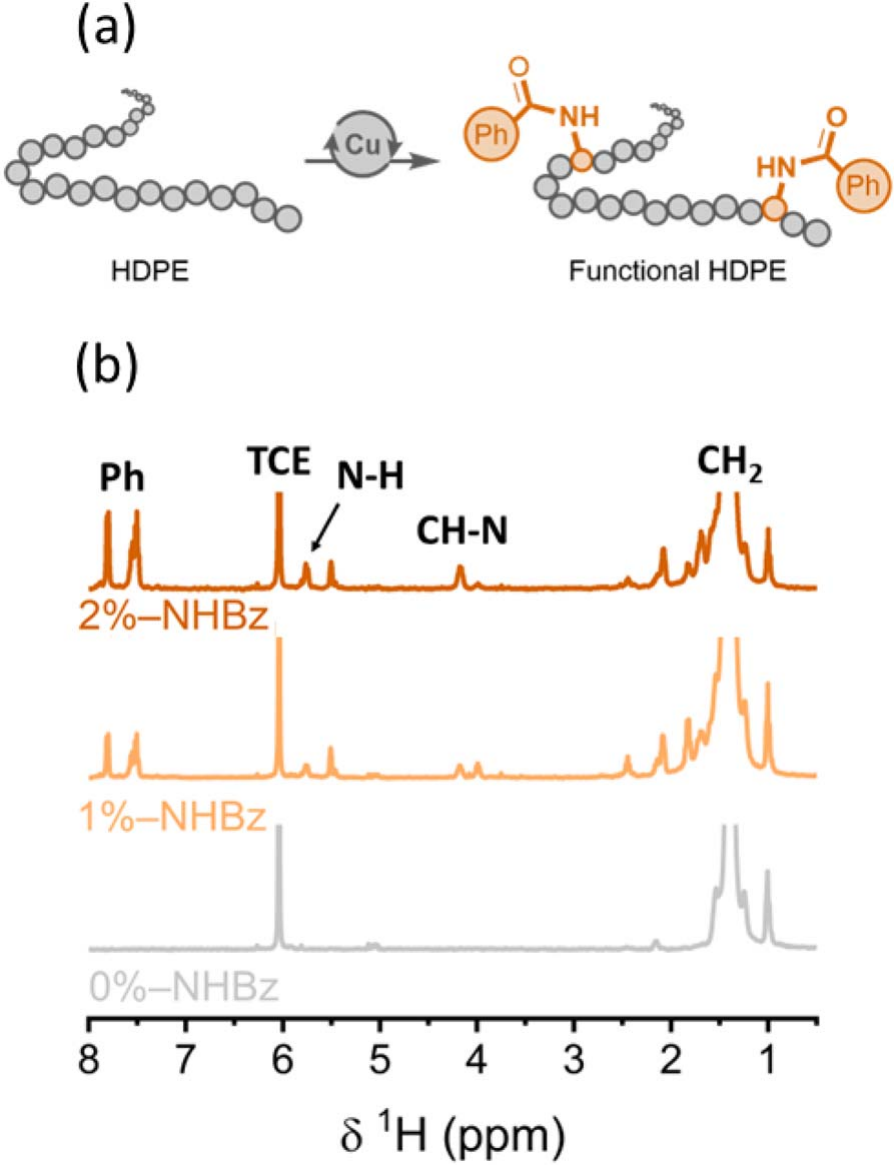
(a) Amination of high-density polyethylenes (HDPE) by a copper catalyst. Conditions: 1 equiv. C_2_H_4_ units, 0.1 mol% (C_8_-phen)CuCl_2_, 4–10 mol% benzamide, 8–20 mol% *t*BuOO*t*Bu, 1,2-dichlorobenzene, 120 °C. C_8_-phen = 3,8-dioctyl-1,10-phenanthroline. (b) ^1^H NMR spectra of HDPEs with 0, 1, and 2 mol% benzamide incorporation (gray, light orange, and dark orange, respectively) acquired at 100 °C at 9.4 T in deuterated tetrachloroethane (TCE).

Recently, we reported the catalytic amidation of polyethylene to create a series of amide-modified polyethylenes with enhanced mechanical properties (Fig. 1a).^41^ Given that this method incorporates controllable amounts of various amides onto the polymers and that the chemical shifts of the protons on the amides are distinct from those of the protons on the aliphatic polymer chains, we envisioned that NMR relaxometry measurements of a systematic series of these amide-containing polyethylenes would lead to new insights into the structure of functional polyolefins.

Here, we show, by a combination of solid-state NMR relaxometry, calorimetry measurements, and X-ray scattering experiments, that covalent attachment of low percentages of benzamidyl groups to high-density polyethylene (HDPE) decreases the crystallinity of the material and increases the mobility of the polymer chains. While such a decrease in crystallinity is expected upon introduction of large polar groups, our NMR analysis of the molecular mobility of both polymer chains and amidyl groups, in combination with X-ray scattering experiments, reveals that the polar amide groups reside in the RAF between the crystalline and the amorphous regions, leading to decreases in crystalline phase thickness and stronger interactions between the amorphous and crystalline phases. The insights from this work illuminate factors that impact the bulk mechanical properties of amide-containing polyethylenes and the relationships between polymer composition and microstructure in these newly accessible materials.

## Results and discussion

### Effect of amidyl groups on the crystallinity of polyethylenes

**Fig. 2:**
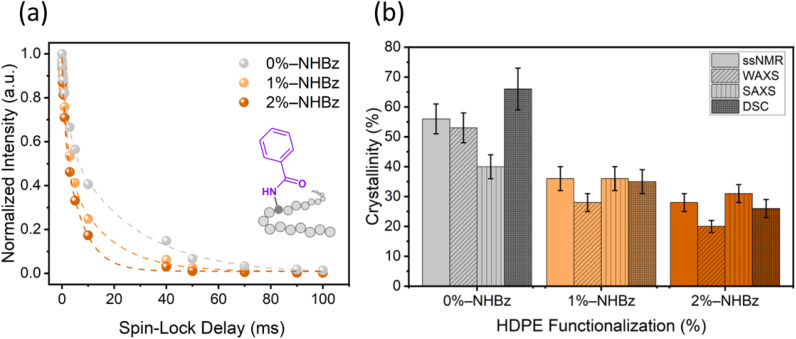
(a) Normalized ^1^H *T*_1ρ_ intensity decay curves of HDPEs with 0, 1, and 2 mol% benzamide incorporation (gray, light orange, and dark orange, respectively) as a function of spin-lock delay time. Data were acquired at 9.4 T with spin-lock field of 150 kHz. (b) Percentage of crystallinity in benzamide-containing HDPEs calculated from ssNMR ^1^H *T*_1ρ_ measurements (solid bar), WAXS measurements (diagonal lines in bar), SAXS measurements (striped bar), and DSC measurements (dark color). Error bars represent standard deviation.

To understand the effect of functional groups on the microstructure, and ultimately the properties, of polyethylene, we synthesized a series of amide-functionalized HDPEs that contain 0–2 mol% amidyl groups, relative to monomer units, and analyzed these polyethylene films by a series of quantitative methods that assess crystallinity. The degree of amide incorporation was determined by the intensity of the resonance from the methine proton α to the amidyl group (^1^H NMR chemical shift = 4.12 ppm for benzamide), *versus* that of the resonance from unmodified methylene units, by ^1^H NMR spectroscopy (Fig. 1b). The degree of crystallinity of the unmodified and amide-containing HDPE films was first analyzed by ^1^H spin-lattice relaxation in the rotating frame (*T*_1ρ_), a sensitive solid-state NMR technique for probing molecular motion in the crystalline, rigid regime of the polymers.^42^ The respective decay curves of ^1^H *T*_1ρ_ intensity are shown in Fig. 2a. A three-component, exponential fit was applied to these data to deconvolve the amorphous, interphase, and crystalline phases of the polymer.^33,35,36^ Tightly packed chains in the crystalline region are less mobile than chains in the amorphous region; thus, we assigned the longest *T*_1ρ_ value, corresponding to the slowest relaxation rate, to the rigid, crystalline region of the sample. The nominal percentage (%) of the crystalline region was calculated for each sample and found to be 56%, 36%, and 28% for 0, 1, and 2 mol% HDPE respectively, revealing a decrease in overall crystallinity with increasing amide incorporation (see Table S9 for the percentages of all components).

To complement these ssNMR relaxometry measurements, we conducted wide-angle X-ray scattering (WAXS), small-angle X-ray scattering (SAXS), and differential scanning calorimetry (DSC) experiments to gain independent values of the percentage crystallinity of these HDPE materials (Fig. S4 and S30). Fig. 2b summarizes our quantitative analysis of the percentage of crystalline domains in the polyethylene samples, as determined by ssNMR, WAXS, SAXS, and DSC (nominal percentages are summarized in Table S11). Some variations between the calculated percentages of crystallinity were observed, and these variations likely result from the different proxies for crystallinity used in these methods (*i.e.*, chain mobility, ordering in crystal structure, or enthalpy). Yet, these comparisons show clearly that the percentage of crystallinity decreases monotonically as the degree of amide incorporation increases.

WAXS and SAXS measurements also enabled determination of the crystal size and the crystalline thickness, accordingly (Fig. S30). These data showed that the crystal size and the crystalline thickness both decrease with increasing benzamide incorporation (Fig. S32). To corroborate these calculations, the crystal size was determined from proton spin diffusion (see Fig. S33 and S34 for discussion and calculations), and these values were similar to those determined from the X-ray data. Together, these results clearly show that the addition of amidyl groups to the polymer leads to decreases in the size of the crystallites and increases in the size of the amorphous phase. Based on these results, we conclude that the amidyl groups do not reside in the crystalline phase; this conclusion aligns with previous studies showing that large groups appended to polyethylene are located outside the crystalline domain.^11,12,18^

### Location of amidyl groups within semicrystalline polyethylenes

While it is clear that the amidyl groups are excluded from the crystallites, we sought to determine more precisely the position of the functional groups within the amorphous regions. Prior work showed that ^13^C relaxation measurements could be used to determine the location of branches within the crystalline and amorphous phases of methyl- and ethyl-branched polyethylenes;^43–46^ however, the low levels of amides in our materials precluded the application of ^13^C measurements because of low signal intensity; therefore, we employed ^1^H *T*_1ρ_ measurements to determine the proton mobility of our samples.

**Fig. 3:**
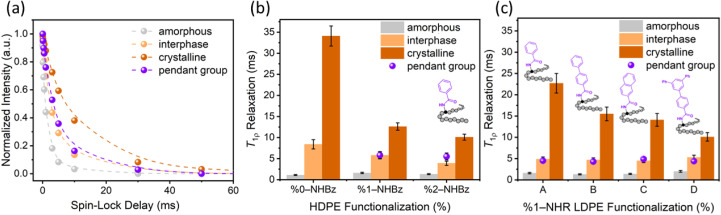
(a) Intensity decay curves as a function of spin-lock delay time for 1%-NHBz HDPE, determined by deconvolution of the respective ^1^H *T*_1ρ_ spectra. Data were acquired at 9.4 T with spin-lock field of 150 kHz. ^1^H *T*_1ρ_ relaxation rates of each component of (b) functionalized HDPEs (0–2 mol%) containing benzamidyl groups, and (c) functionalized LDPEs (1 mol%) containing benzamidyl (A), 4-phenyl-benzamidyl (B), 2-naphthamidyl (C), and 4-(3,5-diphenylphenyl)-benzamidyl (D) groups. Phase color coding: amorphous (gray), interphase (light orange), crystalline (dark orange) and pendant group (purple).

Specifically, ^1^H *T*_1ρ_ spectra in the rotating frame at each spin-lock delay time were deconvolved and separated into four groups of differing molecular mobility: the methylene resonance of the crystalline region, the amorphous region, and the interphasial region (rigid amorphous fraction (RAF)), and the aryl resonance of the functional group (resonating between 7.5–8 ppm). The slowest motion (10–35 ms) was attributed to the crystalline domain, the fastest motion (∼1 ms) was attributed to the amorphous domain, and the intermediate motion was attributed to the interphase domain (4–8 ms). A representative spectrum can be found in Fig. S35 and S36. The intensity of each separate peak was plotted at each spin-lock delay timepoint. Fig. 3a shows a representative example of the ^1^H intensity decay curves for the separate groups of HDPE containing 1 mol% amide incorporation, and Fig. 3b summarizes the ^1^H *T*_1ρ_ relaxation data, determined by fitting each decay to a mono-exponential curve for each unmodified and functionalized HDPE sample. Based on these measurements, we observed that the ^1^H *T*_1ρ_ relaxation times decrease with increasing levels of functionalization, suggesting that the sizes of crystallites decrease accordingly. The mobilities of the amorphous regions of the three polymer samples were comparable (1.1–1.6 ms). We found that the ^1^H *T*_1ρ_ relaxation times of the pendant amidyl groups are similar to those of the interphasial region (RAF) in each case, suggesting that the mobilities of these two components of the polymer are similar and that they are in close proximity.

This phenomenon has been observed in polyethylenes containing aliphatic branches;^11,25,43,47^ small aliphatic pendants, such as methyl groups, have been shown to reside in the interphase, determined by ^13^C *T*_1_ and *T*_2_ analyses, whereas larger aliphatic pendants, such as ethyl and butyl groups, appear to reside in the mobile, amorphous phase, as determined by short *T*_1ρ_ relaxation times.^46,48,49^ During the preparation of this manuscript, small oxo groups also have been shown by ^1^H and ^13^C NMR spectroscopy and relaxometry to reside predominantly in the interphasial region of HDPE.^50^ Our NMR data, together with the SAXS data, support the conclusion that the amidyl groups accumulate at the phase boundary of the crystalline regions, despite what one might predict from the large size of the amidyl group, and, thus, they are localized in the RAF.

To confirm that the similar ^1^H *T*_1ρ_ relaxation values for the amides and for the methylene groups in the interphasial regions were due to colocalization, rather than inherently similar relaxation times, we measured the ^1^H *T*_1ρ_ relaxation times of small-molecule model compounds containing amides in the solid phase (Table S13). We found that the ^1^H *T*_1ρ_ values for relaxation of amides bound to small alkanes are significantly longer (12–30 ms) than the ^1^H *T*_1ρ_ values for the amides bound to polyethylene (∼6 ms). This difference supports our conclusion that the similarity between the mobilities of the functional groups and the interphasial region of the polymer is best attributed to colocalization.

We next analyzed a series of low-density polyethylene (LDPE) materials containing a variety of different amides to investigate the effect of the structure of the amide on the location of such groups (Fig. 3c). We varied the size of the amides to determine the steric effects of the pendant groups on the microstructure of the polymer. As shown in the figure, analysis of the ^1^H *T*_1ρ_ relaxation times of the functionalized LDPEs suggests that these amidyl groups also are localized in the interphasial region because the local mobility of these groups and the methylene groups in this region were similar to each other and were distinct from those of the methylene groups in the crystalline and amorphous regions. The mobility of the methylene groups within the crystalline region decreased with increased size of the aryl group attached to the amide, suggesting that these materials possess smaller crystalline domains.

**Fig. 4:**
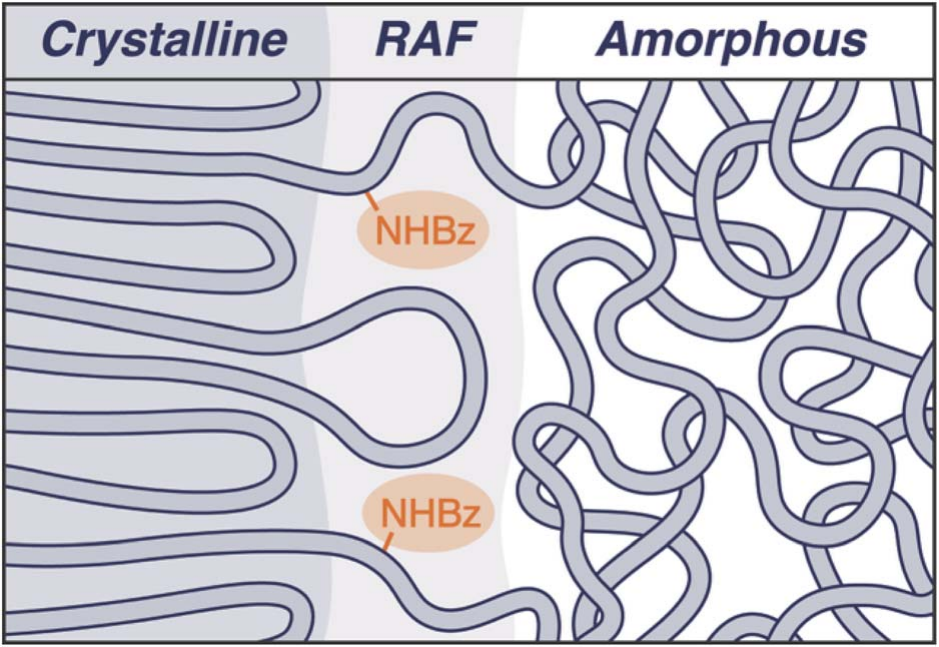
Schematic representation of the microstructure of the amide-containing polyethylenes, portraying the location of the benzamidyl groups within the RAF (rigid amorphous fraction).

RAF formation is influenced by many factors, including the crystallization cooling rate, polymer chain length, architecture, and flexibility of the chains.^25,27^Fig. 4 shows a schematic representation of the RAF in the amide-containing polyethylenes; we envision that the amides shorten the segments that can crystallize and, thus, are excluded from the crystallites but remain positioned at the phase boundary (RAF). It has been shown previously that frequent crossing of the polymer chains between the amorphous and crystalline domains, occurring at the phase boundary (RAF), results in strong coupling of the phases and leads to enhanced mechanical responses.^25,27,32,51^ Given these prior findings, we hypothesize that the accumulation of amidyl groups within the RAF could impact chain mobility between domains.

### Effect of amidyl groups on polymer chain mobility

**Fig. 5:**
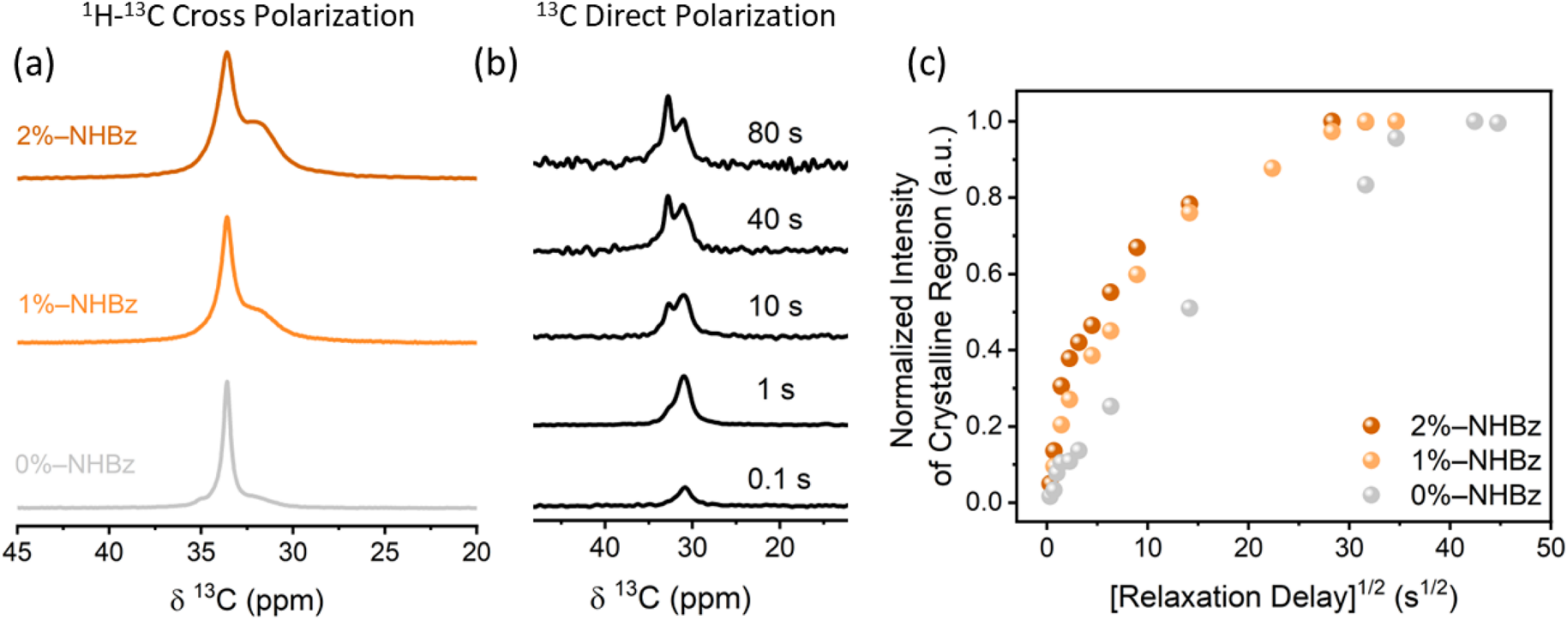
(a) ^1^H–^13^C CP measurements of HDPEs with 0, 1, and 2 mol% benzamide incorporation (gray, light orange, and dark orange, respectively) highlighting a distinct amorphous signal (∼31 ppm) and a distinct crystalline signal (∼33 ppm). Spectra were acquired at 11.75 T with contact time of 1 ms and spinning speed of 3.5 kHz. (b) ^13^C direct polarization measurements of unmodified HDPE with different exchange times (recovery time values). (c) Normalized ^13^C intensity of the crystalline peak of HDPEs with 0, 1, and 2 mol% benzamide incorporation (gray, light orange, and dark orange, respectively) plotted as a function of the square root of the relaxation delay (*d*_1_).

To test this hypothesis, we employed ^13^C NMR spectroscopy to explore the local chain mobility of each domain in the functionalized samples and to evaluate the effect of functional groups on the exchange of chain segments between the domains. Fig. 5 shows line shapes and ^1^H–^13^C cross polarization (CP) dynamics of the unmodified and functionalized HDPE samples. The isotropic chemical shift from these measurements was used to identify ^13^C peaks from the different phase domains; the amorphous peak of polyethylene resonates at ∼31.1 ppm and the crystalline peak resonates at ∼33 ppm.^52^ Although peak intensity is not quantitative in CP spectra, the intensity of the amorphous peak does clearly increase with increased levels of amide-incorporation, suggesting a large change in the CP dynamics. We measured the CP contact time dynamics for each polymer sample (Fig. S37) and extracted the time constant for the increase in intensity of the crystalline peak. These time constants were found to be 1200 µs, 800 µs, and 650 µs for polyethylenes containing 0, 1, and 2 mol% benzamidyl groups, respectively (see Fig. S37). These time constants are governed by local proton-carbon dipolar fields, which diminish with increasing molecular mobility on the CP timescale. That is, short time constants are consistent with decreased local mobility. Therefore, our results indicate that the carbon mobility in the crystalline domains of HDPEs decreases with increasing amounts of amidyl groups in the RAF.^45^ Furthermore, we observed that the linewidth of the crystalline peak of the amide-containing HDPE was ∼100 Hz greater than that of the unmodified HDPE. We hypothesize that this broadening results from a decrease in *T*_2_ relaxation or an increase in heterogeneity of sites resulting from the disruption of the crystalline domain by the functional groups.

It has previously been shown that three distinguished chain motions are present in polyethylenes, namely α, β, and γ relaxations, which combine both translational and rotational motion.^53–55^ Helical jumps, consisting of the motion of chain segments between the various regions, are correlated with α relaxation and were previously shown by ssNMR measurements to occur on the timescale of seconds at room temperature.^55–57^ Given that the amidyl groups disrupt the crystalline domain, we hypothesize that the interdomain diffusion of chains between the amorphous and crystalline regions and their influence on one another will be different for the modified and unmodified polymers.

To test this hypothesis, we conducted a quantitative analysis by ^13^C direct polarization measurements. The substantial differences in *T*_1_ relaxation times of chains within the crystalline and amorphous regions (see Table S12) enabled us to monitor chain diffusion between the two regions by ^13^C NMR spectroscopy.^56,57^ Specifically, this experiment consisted of an initial saturation of the NMR signal, followed by increasing variation of the recovery delay (exchange time, Fig. 5b). Diffusion of short-relaxing chains into domains of longer-relaxing chains will be manifest as the appearance of ^13^C signal prior to that expected from relaxation behavior alone. In the absence of chain diffusion and at short recovery times (ms to s), no signal from the crystalline region is expected, due to its extremely long *T*_1_ relaxation time. However, the opposite is apparent in our data; we observed a signal from the crystalline region after a *T*_1_ delay of only 1 s, which is significantly shorter than the *T*_1_ relaxation value for this region (longer than 400 s, Table S12). This observation implies that the emergence of the crystalline peak originates from a phenomenon besides spin-lattice relaxation. Therefore, we conclude that translational motion of the polarized amorphous chains occurs into the crystalline region and that this motion creates a change in chemical shift, leading to the resulting peak observed for the crystalline chains.^56,57^

We next assessed the degree of translational motion of chains in the amorphous region into the crystalline region, as a function of the amount of benzamide functionality.^54^Fig. 5c shows the normalized ^13^C crystalline peak intensity as a function of the square root of the relaxation delay, *d*_1_ (see Fig. S36 for ^13^C spectra of the polymer samples). This analysis enables direct comparison of the diffusion rates of each sample by comparing the linearized slope of the peak intensities at short delays (0–30 s). The crystalline peak of 2%-NHBz HDPE grows more rapidly than the crystalline peak of both the unmodified HDPE and the 1%-NHBz HDPE, suggesting that the rate of diffusion of chains from the amorphous regions into the crystalline region increases with increasing amide content. At longer recovery times (>30 s), peak growth of each sample slows and is dominated by *T*_1_ relaxation of the carbon atoms in the crystalline region. These observations show that the addition of amidyl groups leads to enhanced interdomain dynamics. Such effects likely impact the bulk mechanical properties of the materials.

### Impact of microstructural changes on the mechanical properties of polyethylenes

**Fig. 6:**
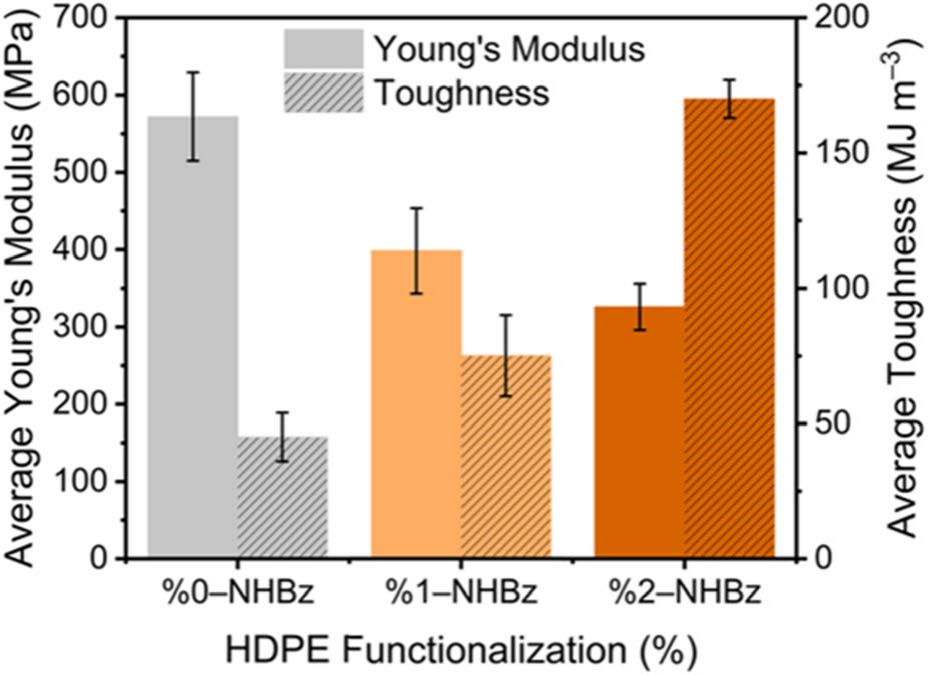
Average Young's Modulus (solid bar) and average toughness (diagonal lines in bar) of HDPE and benzamide-containing HDPEs determined from tensile tests. Error bars represent standard deviation.

To illustrate these effects, we measured the bulk mechanical properties of the amide-containing polymers (Fig. 6). Tensile testing of HDPEs containing 0%, 1%, and 2% of amidyl units (Fig. S1–S3) showed that the average toughness of the polymers increased with increasing levels of amide, while the average Young's Modulus decreased; similar phenomena have been observed previously upon introduction of other large polar groups onto polyolefins.^6,7^ Our detailed NMR analysis reveals that the specific location of these pendant groups within the RAF leads to the enhanced phase coupling that affects these mechanical properties; given that the results of these tensile tests align with the expected trend after addition of short chain-branching onto the polymer backbone,^6,41,58^ our findings also could provide insights into the properties of other functional polyolefins more generally. Finally, analysis of the polymer films that had undergone tensile testing revealed that the mobility of the functional groups remains most similar to that of the slow-moving chains, suggesting that the amides remain within a phase boundary, even after stretching (see Fig. S39 for details).

## Conclusions

In summary, we reveal insights into the microstructure of amide-modified polyethylenes by NMR spectroscopy and relaxometry, X-ray scattering, and calorimetry. Like other large pendant functional groups, the amidyl groups in these materials are excluded from crystallites and reduce overall crystallinity. In contrast to conventional techniques that analyze crystallinity, such as X-ray scattering and calorimetry, our NMR studies, based on spin relaxation, enable analysis of microstructure with precise molecular specificity; the results from these studies suggest that the polar amidyl groups reside specifically in the interphase (RAF) between the crystalline and the amorphous regions. Given the similarity between amides and other large pendant groups, such findings should help locate other functional groups in functionalized polyolefins. The incorporation of these groups within the RAF leads to increased inter-domain chain mobility between the amorphous and crystalline regions, and, as a result, leads to changes in bulk material properties. Specifically, polymers with faster chain diffusion between morphological regions exhibited higher average toughness and reduced stiffness. Utilization of NMR to pinpoint the location of the benzamidyl groups within the microstructure of HDPE and to reveal the local and inter-domain chain mobility sheds light on factors that affect the properties of functional polyolefins and provides detailed insights into the microstructures of these newly accessible materials.

## Author contributions

S. H. and N. R. C. contributed equally to this work. S. H., N. R. C., B. A. H., J. F. H., and J. A. R. conceptualized this project. S. H., N. R. C., and J. A. R. contributed to the design of the project. N. R. C. synthesized and prepared all polymer and small molecule materials, conducted DSC, GPC, and NMR characterization, and conducted tensile testing of the materials. Z. P., F. Y., M. H. and C. W. designed the experiments and performed all X-ray measurements and analyses. NMR characterization and analysis were performed by S. H. and J. I. Further NMR methodology and development was performed by S. H. and R. G. Spin diffusion simulations were performed by S. N. F. The original draft was written by S. H., N. R. C., J. F. H., and J. A. R. All authors contributed to the final draft and editing. J. A. R. and J. F. H. supervised research, provided project administration, and acquired funding along with B. A. H.

## Conflicts of interest

B. A. H. has a financial interest in Cyklos Materials and Sepion Technologies. The other authors declare that they have no competing interests.

## Supplementary Material

SC-017-D5SC08878J-s001

## Data Availability

All data needed to evaluate the conclusions are present in the paper and/or the supporting information (SI), as well as in the Dryad Repository at https://doi.org/10.5061/dryad.kwh70rzk3. Supplementary information: experimental details of the methods presented in the text, ^1^H NMR spectra determining the level of incorporation of the functional groups, synthesis of amide-containing polyethylenes, synthesis of small molecule models, synthesis of amides, mechanical and thermal properties of the polymers, ^1^H and ^13^C NMR, GPC, and tensile testing characterization of the materials, correlation function for SAXS data, ^1^H spin diffusion simulation, WAXS *q* values and ^13^C *T*_1_ relaxation values for the polymer materials, ^1^H–^13^C build up curves, and ^1^H *T*_1ρ_ for the small molecule model and stretched polymer materials. See DOI: https://doi.org/10.1039/d5sc08878j.
