# Supplementary material for: Microstructure of amide-functionalized polyethylenes determined by NMR relaxometry
Source: Chem Sci. 2026 Jan 29;17(12):5984–92. doi: 10.1039/d5sc08878j (PMC12853295; doi:10.1039/d5sc08878j)
Supplement: SC-017-D5SC08878J-s001 [file SC-017-D5SC08878J-s001.pdf]

Supplementary Information (SI) for Chemical Science.
This journal is © The Royal Society of Chemistry 2026

Supporting Information for:

Microstructure of Amide-Functionalized Polyethylenes Determined by NMR Relaxometry

Shira Haber,[1,9+*] Nicodemo R. Ciccia,[2,3,10+] Zhengxing Peng,[1,4] Feipeng Yang,[4,5] Julia Im,[6] Mutian Hua,[1,11] Sophia N. Fricke,[6,7] Raynald Giovine, [7] Brett A. Helms,[1,8] Cheng Wang,[4] John F. Hartwig,[2,3*] and Jeffrey A. Reimer[1,6*]

[1] Materials Sciences Division, Lawrence Berkeley National Laboratory, 1 Cyclotron Road, Berkeley, CA 94720, USA.

[2] Department of Chemistry, University of California, Berkeley, Berkeley, CA 94720, USA and Division of Chemical Sciences Lawrence Berkeley National Laboratory, Berkeley, CA 94720, USA.

[3] Division of Chemical Sciences, Lawrence Berkeley National Laboratory, Berkeley, CA 94720, USA.

[4] Advanced Light Source, Lawrence Berkeley National Laboratory, 1 Cyclotron Road, Berkeley, CA 94720, USA.

[5] National Synchrotron Light Source II, Brookhaven National Laboratories, Upton, NY 11973, USA.

[6] Department of Chemical and Biomolecular Engineering, University of California Berkeley, Berkeley, CA 94720, USA.

[7] Pines Magnetic Resonance Center, Core NMR Facility, College of Chemistry, University of California, Berkeley, Berkeley, CA 94720, USA.

[8] The Molecular Foundry, Lawrence Berkeley National Laboratory, Berkeley, CA 94720, USA.

[9] Department of Chemistry, Bar-Ilan University, Ramat-Gan 5290002, Israel.

[10] Department of Chemistry, Princeton University, Princeton, New Jersey 08544, USA.

[11] School of Environmental and Forest Sciences, University of Washington, Seattle, WA 98195, USA.

[+] co-first authors   *corresponding authors

This file includes:

Experimental details

Scheme S1

Figures S1 – S39

Tables S1 – S13

1. Experimental Details

All reactions were conducted under air unless otherwise specified. *N*-cyclohexylbenzamide,[1] *N*-octylbenzamide,[1] (phen)$CuCl_2$ [phen = 1,10-phenanthroline],[1] and (C_8-phen)$CuCl_2$ [C_8-phen = 3,8-dioctyl-1,10-phenanthroline][1] were prepared according to previously reported procedures. All other materials were purchased from commercial suppliers and used as received unless otherwise noted. High density polyethylene (HDPE) was purchased from Sigma-Aldrich (427985-1KG). Low density polyethylene (LDPE) was purchased from Sigma-Aldrich (428043-1KG). Polymer films were prepared by melt-pressing using at 120 °C – 140 °C to afford films with a thickness of 200±10 μm.

1.1 Solution-state NMR

Solution-state nuclear magnetic resonance spectra (NMR) measurements of ^{1}H and ^{13}C nuclei were obtained on Bruker 400 (9.4 T), 500 (11.75 T), 600 (14.1 T), and 700 (16.44 T) MHz spectrometers at the Pines Magnetic Resonance Center at the University of California, Berkeley. Experiments were conducted at 100 °C – 120 °C for polymer samples and at 25 °C for small molecule samples. Chemical shifts are reported in ppm (δ) and referenced against the resonance of residual solvent (^{1}H NMR: $CDCl_3$, 7.26 ppm; $C_2D_2Cl_4$, 6.00 ppm; ^{13}C NMR: $CDCl_3$, 77.16 ppm; $C_2D_2Cl_4$, 73.78 ppm). Spin-spin couplings are described as singlet (s), doublet (d), triplet (t), quartet (q), quintet (quint), broad (br) or multiplet (m), with coupling constants (J) in Hz. zg30 Bruker pulse program was used with relaxation time of 1–5 s and 16 scans. For ^{13}C measurements, zgpg30 Bruker pulse program was used with relaxation time of 1 s with 512 scans. RF fields were 16.67 kHz and 29 kHz for ^{1}H and ^{13}C, respectively.

1.2 Solid-state NMR

^{1}H solid-state NMR (ssNMR) experiments on a 400 MHz (9.4 T) using a Bruker BioSpin spectrometer equipped with an Avance IV Neo console with a 1.3 mm double resonance HX magic angle spinning (MAS) probe. Samples were loaded in 1.3 mm zirconia rotors, closed using Vespel® caps, and spun at the magic angle at spinning speeds of 60 kHz using dry nitrogen.

^{1}H ssNMR spectra were obtained using a rotor synchronized Hahn echo sequence ($90° - \tau_R - 180° - \tau_R -$ AQ) with an RF field of 119 kHz. T_1 relaxation measurements were acquired using a saturation recovery pulse sequence followed by an echo detection. The initial saturation of ^{1}H magnetization was achieved using a train of 100 short 90° pulses (119 kHz) spaced by 5 ms. Relaxation time $T_{1\rho}$ was measured at room temperature with a standard spin-lock experiment (90° pulse immediately followed by a phase shifted variable-length spin-locking pulse) with RF spin-lock field of 150 kHz. Crystallinity was calculated by fitting the ^{1}H $T_{1\rho}$ data to a three-exponent fit (assuming the polymer

consists of amorphous, crystalline, and interphasial regions). The mobility of the crystalline region was attributed to the longest $T_{1\rho}$ motion and the slowest mobility and calculated in the following way:

$$\chi_c = \frac{A_c}{A_c+A_I+A_{NC}} \quad (1)$$

where A_C is the area of the longest $T_{1\rho}$ relaxation time (crystalline), A_I is the area of the moderate $T_{1\rho}$ relaxation time (interphase), and A_{NC} is the area of the shortest $T_{1\rho}$ relaxation time (amorphous).

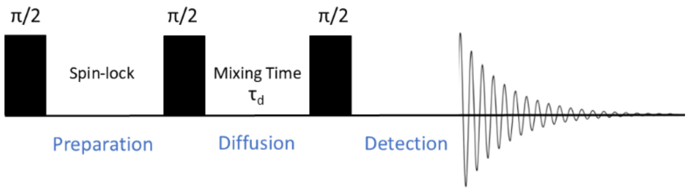

Scheme S1. Schematic representation of the modified spin-diffusion pulse sequence.

Modified spin-diffusion experiments were conducted following a previously reported procedure.[2] As illustrated in Scheme S1, a 90° pulse was applied, followed by a spin-lock for 8–12 ms. This spin-lock period is the preparation stage that removes short $T_{1\rho}$ components. After another 90° pulse, a varying delay time (ranging from 1–1500 ms for unmodified HDPE or 1–1000 ms for 2%–NHBz HDPE) was applied to allow spin-spin diffusion. After this delay, τ_d, a final 90° pulse was applied, and the signal was detected after varying delay times (signal detection was conducted in a manner similar to that of a standard $T_{1\rho}$ measurement). The data were fit to a multi-exponent decay; the shortest $T_{1\rho}$ motion was attributed to the amorphous phase, and the longest $T_{1\rho}$ motion was attributed to the crystalline phase.

Numerical simulations were performed using MatLab (Mathworks, Natick, MA) according to an approach described previously.[2] In this model, the geometry of the crystallite is chosen to be a periodic array of planar crystallites of infinite two-dimensional length with finite thickness in the third dimension. The spin energy transport equation to be solved is:

$$\frac{\partial M(x,t)}{\partial t} = \nabla \cdot [D(x)\nabla M(x,t)] - \frac{1}{T(x)}\left[M(x,t) - M_{eq}\right] \quad (2)$$

where $M(x,t)$ is the nuclear magnetization density as a function of position, x, and time, t, $D(x)$ is the local diffusion coefficient for spin energy, $\frac{1}{T(x)}$ is the local intrinsic spin-lattice relaxation rate, and M_{eq} is the equilibrium magnetization. Because the proton magnetization is spin-locked during the preparation and detection steps, M_{eq} is very close to zero and $T = T_{1\rho}$, which is measured experimentally. The optimal fit of the model is determined by varying the crystallite size and mass fraction parameters to find the best agreement with the spin-diffusion data. Here, the diffusion coefficient value of $D = 5 \cdot 10^{-12}$ cm^2 s^{-1} was used.[2]

Additional ^1H and ^{13}C ssNMR measurements were recorded on a 500 MHz (11.75 T) using a Bruker BioSpin spectrometer equipped with an Avance-I console and 4.0 mm double resonance HX magic angle

spinning (MAS) probes that tune to both ^{1}H and ^{13}C. Samples were loaded in 4.0 mm zirconia rotors, with a layer of Teflon tape below and over them to assist in spinning stability, closed using Kel-F® caps, and spun at the magic angle with spinning speed of 3.5 kHz using dry nitrogen.

^{13}C cross polarization (CP) experiments were executed under the Hartmann-Hahn matching condition with a relaxation delay of 2 s, and varying contact periods between 0.5 and 4 ms. During ^{13}C acquisition, high-power ^{1}H decoupling was applied using 28 kHz SPINAL-64[3] (Small Phase Incremental Alternation with 64 steps) decoupling scheme. ^{13}C direct excitation experiments were conducted in a similar manner to T_1 measurements with a saturation recovery pulse sequence followed by detection. The initial saturation of ^{13}C magnetization was achieved using a train of 100 short 90° pulses (56 kHz) spaced by 1 ms. RF fields of 59 kHz and 56 kHz were used for ^{1}H and ^{13}C, respectively.

^{1}H and ^{13}C chemical shift were referenced with respect to tetramethylsilane using the CH_2 resonance of adamantane as a secondary external reference at ^{13}C = 38.48 ppm and ^{1}H = 1.8 ppm. All solution and solid-state NMR data were processed using Bruker TopSpin 4.1.4 and 4.3.0 and DMfit software.[4]

1.3 High-temperature size exclusion chromatography

High-temperature size exclusion chromatography (HTSEC) was performed on a Tosoh EcoSEC-HT with three TSKgel GMHhr-H(S) HT columns in series. Runs were performed with 1,2,4-trichlorobenzene + 0.05% butylated hydroxytoluene (BHT) as a mobile phase at 135 °C and 1 mL/min. Relative molecular weight was determined relative to polyethylene standards.

1.4 Differential scanning calorimetry

DSC was performed on a TA Discovery DSC 25 instrument. Each sample (ca. 5 mg) was placed in a hermitic aluminum pan, sealed, and scanned at a rate of 10 °C/min from 0 °C to 170 °C. Data were plotted from the second heating cycle. Peak melting temperatures (T_m) were recorded on the second scan. The relative heat flow was normalized with respect to the sample mass and the enthalpy of melting (ΔH_m) was obtained by integration of the melt peak. Measurements were conducted with at least 3 samples and average values are reported.

Percent crystallinity (X_c) was calculated using the following equation:

$$X_c = 100 * \frac{\Delta H_m}{\Delta H_{100}} \quad (3)$$

ΔH_m is the enthalpy absorbed during heating, and ΔH_{100} is the enthalpy absorbed during heating of a sample that is 100% crystalline. For polyethylene, ΔH_{100} = 293 J/g.[5]

The average percent crystallinity (X_c) for each sample was calculated using the equation above with the average ΔH_m value for that sample. The uncertainty of the average percent crystallinity (∂X_c) was calculated using the following equation:

$$\partial X_c = \frac{100}{\Delta H_{100}} * \partial \Delta H_m \quad (4)$$

1.5 Tensile testing

Preparation of polymer films. Polymer films were prepared on a hot press at 140 °C (HDPE samples) or 120 °C (LDPE samples) for 90 seconds to provide melts. Specifically, polymer samples between two Kapton films were pressed between steel plates at 2000 psi. Teflon shims were used to control film thickness. The samples were then cooled at room temperature to provide films (200±10 μm).

Polymer films (200±10 μm) were cut into a dog-bone geometry using a cutting die (ASTM D-638-V) to obtain samples that were 9.53 mm in length and 3.18 mm in width. Tensile testing was conducted according to ASTM D638 on an Instron universal materials tester. Tensile stress and strain were measured at room temperature using an extension rate of 50 mm/min. Measurements were repeated for at least four samples, and average values are reported.

1.6 Wide-angle x-ray scattering (WAXS) and Small-angle x-ray scattering (SAXS)

WAXS measurements were conducted at the Complex Materials Scattering beamline (CMS, 11-BM) at NSLS-II, BNL. Polymer films were mounted on the transmission stage, aligned perpendicular to the direction of the incident beam. X-ray was transmitted through a hole on the sample holder and directly onto the free-standing polymer film. The scattering patterns were collected with an in-vacuum detector (DECTRIS Pilatus 800K), equipped with a pixel size of 172 μm and placed 260 mm away from the sample for WAXS and 5.03 m away for SAXS. The experiments were conducted with a 13.5 keV X-ray beam with a wavelength of 0.9184 Å and an exposure time of 5 s for each scattering pattern. The 2D scattering patterns were reduced to 1D intensity (I) vs. the wave vector transfer (q) profiles by the code at beamline 11-BM. Quantitative analysis was conducted with Nika based on Igor,[6] with peak fitting with Gaussian, for WAXS data. The correlation function from SAXS data was analyzed with SASview 6.0.0 (www.sasview.org/).

The percentage of the crystalline domain, crystallinity χ_c, from the WAXS data, was calculated using equation (5):[7,8]

$$\chi_c = \frac{A_c}{A_a + A_c} \quad (5)$$

where A_a is the peak area of the amorphous halo at ~1.4 $Å^{-1}$ and A_c is the peak area of the crystalline peaks. The crystal size was estimated with Sherrer equation[9] which accounts for the broadening of the (110) peak due to finite crystal size according to equation (6):

$$L_{(110)} = \frac{2\pi K}{\Delta q} \quad (6)$$

where K is a shape factor taken as 0.9, Δq is peak width which is taken as the Full Width at Half Maximum (FWHM). The crystal size along *a* is estimated from (200) peak and the crystal size along *b* is estimated from (110) peak.

The percentage of the crystalline domain, crystallinity χ_c, from SAXS data, was calculated using the following equation (7):[10]

$$\chi_c = \frac{d_c}{d_a + d_c} \quad (7)$$

where d_c is the thickness of the crystalline phase and d_a is the thickness of the amorphous phase.

2. *Determination of the degree of functional group incorporation*

The degree of functional group incorporation was determined by ^1H NMR spectroscopy at 100 °C. The integration of peaks between 1.0 and 1.7 ppm was set to a total of 4 protons (per C_2H_4 unit, integral = 400). The degree of amide incorporation was determined by the relative integration of the resonances from the methine proton α to the amide group (1H, chemical shift = 4.12 ppm for benzamide). The degree of olefin incorporation was determined by the relative integration of the resonances from vinyl protons (2H, chemical shift = 5.46 ppm).

3. *Synthesis of amide-containing polyethylenes*

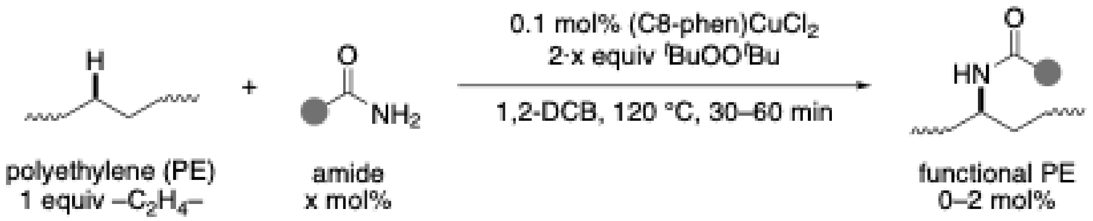

Representative protocol. Amide-modified polyethylenes were synthesized following a modified procedure.[1] Polyethylene (PE) (1.0 g, 35.7 mmol) was dissolved in 1,2-DCB at 120 °C. To the hot solution were added (C_8-phen)$CuCl_2$ (18.8 mg, 0.035 mmol), amide, and ^{t}BuOO^{t}Bu. The reaction mixture was vigorously stirred at 120 °C for the specified time. After this time, while hot, the reaction mixture was poured into 50 mL MeOH while stirring. The precipitated polymer was filtered and collected in a vial. To

purify the material, the polymer was dissolved in minimal amounts of toluene at 120 °C and poured into MeOH. This process was repeated until a pale beige solid was collected. The polymer was dried under vacuum. NMR spectra were collected in $C_2D_2Cl_4$ at 100 °C and GPC traces were collected in 1,2,4-trichlorobenzene at 135 °C.

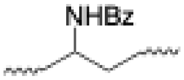

NHBz-HDPE, 1 mol%

1%–NHBz-HDPE: The title compound was prepared according to the representative protocol with HDPE (100 mg, 3.57 mmol, 1 equiv; M_n = 10.8 kg·mol^{-1}, Đ = 5.0), 1,2-DCB (588 µL), (phen)$CuCl_2$ (1.1 mg, 3.5 µmol, 0.1 mol%), benzamide (17.3 mg, 0.143 mmol, 4 mol%), and ^{t}BuOO^{t}Bu (53 µL, 0.290 mmol, 8 mol%) for 1 h. The title compound was isolated as a pale beige powder and melt-pressed into films for subsequent analyses (0.7 mol% -NHBz, 18% yield; M_n = 6.9 kg·mol^{-1}, Đ = 2.8). The ^{1}H NMR spectrum of the title compound matches previously reported data.[1]

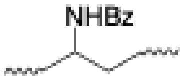

NHBz-HDPE, 2 mol%

2%–NHBz-HDPE: The title compound was prepared according to the representative protocol with HDPE (1.0 g, 35.7 mmol, 1 equiv; M_n = 10.8 kg·mol^{-1}, Đ = 5.0), 1,2-DCB (5.88 mL), (C8-phen)$CuCl_2$ (18.8 mg, 0.035 mmol, 0.1 mol%), benzamide (433 mg, 3.57 mmol, 10 mol%), and ^{t}BuOO^{t}Bu (1.33 mL, 7.28 mmol, 20 mol%) for 30 min. The title compound was isolated as a pale beige powder and melt-pressed into films for subsequent analyses (2.0 mol% -NHBz, 20% yield; M_n = 7.5 kg·mol^{-1}, Đ = 4.0). The ^{1}H NMR spectrum of the title compound matches previously reported data.[1]

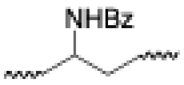

*1%-LDPE-**A***

1%–LDPE-A: The title compound was prepared according to the representative protocol with LDPE (2.0 g, 71.4 mmol, 1 equiv; M_n = 9.5 kg·mol^{-1}, Đ = 6.9), 1,2-DCB (8 mL), C8-phen (31.2 mg, 0.077 mmol, 0.1 mol%), CuI (13.6 mg, 0.071 mmol, 0.1 mol%), benzamide (346 mg, 2.85 mmol, 4 mol%), and ^{t}BuOO^{t}Bu (1.06 mL, 5.8 mmol, 8 mol%) for 1 h. The title compound was isolated as a pale beige powder

and melt-pressed into films for subsequent analyses (1.1 mol% -NHBz, 28% yield; M_n = 7.2 kg·mol^{-1}, $Đ$ = 8.1). The ^{1}H NMR spectrum of the title compound matches previously reported data.[1]

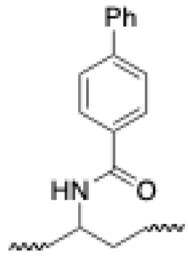

1%-LDPE-B

1%–LDPE-B: The title compound was prepared according to the representative protocol with LDPE (2.0 g, 71.4 mmol, 1 equiv; M_n = 9.5 kg·mol^{-1}, $Đ$ = 6.9), 1,2-DCB (8 mL), C8-phen (31.2 mg, 0.077 mmol, 0.1 mol%), CuI (13.6 mg, 0.071 mmol, 0.1 mol%), 4-phenyl-benzamide (423 mg, 2.14 mmol, 3 mol%), and ^{t}BuOO^{t}Bu (792 µL, 4.33 mmol, 6 mol%) for 1 h. The title compound was isolated as a pale beige powder and melt-pressed into films for subsequent analyses (1.2 mol% -NHR, 40% yield; M_n = 6.7 kg·mol^{-1}, $Đ$ = 8.2). ^{1}H NMR (600 MHz, $C_2D_2Cl_4$) δ 7.88 (d, J = 8.2 Hz), 7.73 (d, J = 8.3 Hz), 7.69 (d, J = 7.8 Hz), 7.54 (t, J = 7.5 Hz), 7.46 (t, J = 7.5 Hz), 5.81 (s), 5.54 – 5.42 (m), 4.32 – 4.12 (m), 2.49 – 2.39 (m), 2.16 – 2.01 (m), 1.77 – 1.64 (m), 1.39 (br), 1.04 – 0.86 (m). ^{13}C NMR (151 MHz, $C_2D_2Cl_4$) δ 208.10, 143.84, 139.98, 130.15, 128.67, 127.71, 127.04, 126.94, 49.94, 37.49, 35.18, 33.90, 33.53, 31.64, 29.89, 29.41, 28.93, 26.66, 25.75, 22.85, 22.35, 13.74.

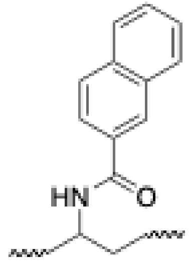

1%-LDPE-C

1%–LDPE-C: The title compound was prepared according to the representative protocol with LDPE (2.0 g, 71.4 mmol, 1 equiv; M_n = 9.5 kg·mol^{-1}, $Đ$ = 6.9), 1,2-DCB (8 mL), C8-phen (31.2 mg, 0.077 mmol, 0.1 mol%), CuI (13.6 mg, 0.071 mmol, 0.1 mol%), 2-naphthamide (245 mg, 1.43 mmol, 2 mol%), and ^{t}BuOO^{t}Bu (528 µL, 2.89 mmol, 4 mol%) for 1 h. The title compound was isolated as a pale beige powder and melt-pressed into films for subsequent analyses (0.8 mol% -NHR, 40% yield; M_n = 7.0 kg·mol^{-1}, $Đ$ = 7.6). ^{1}H NMR (600 MHz, $C_2D_2Cl_4$) δ 8.31 (s), 8.02 – 7.93 (m), 7.87 (d, J = 8.4 Hz), 7.68 – 7.59 (m), 5.90 (d, J = 8.2 Hz), 5.55 – 5.43 (m), 4.33 – 4.13 (m), 2.48 – 2.40 (m), 2.16 – 2.01 (m), 1.81 – 1.67 (m),

1.39 (br), 1.03 – 0.85 (m). ^{13}C NMR (151 MHz, $C_2D_2Cl_4$) δ 140.94, 130.44, 128.89, 128.45, 127.84, 127.54, 127.03, 126.82, 123.71, 50.34, 37.78, 35.50, 34.20, 33.82, 32.43, 31.93, 30.19, 29.70, 29.31, 29.23, 26.96, 26.57, 26.07, 23.14, 22.64, 14.03.

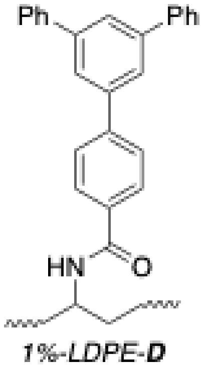

1%-LDPE-D

1%–LDPE-D: The title compound was prepared according to the representative protocol with LDPE (2.0 g, 71.4 mmol, 1 equiv; M_n = 9.5 kg·mol^{-1}, $Đ$ = 6.9), 1,2-DCB (8 mL), C8-phen (31.2 mg, 0.077 mmol, 0.1 mol%), CuI (13.6 mg, 0.071 mmol, 0.1 mol%), 4-(3,5-diphenylphenyl)-benzamide (748 mg, 2.14 mmol, 3 mol%), and ^{t}BuOO^{t}Bu (792 µL, 4.33 mmol, 6 mol%) for 1 h. The title compound was isolated as a pale beige powder and melt-pressed into films for subsequent analyses (1.2 mol% -NHR, 40% yield; M_n = 7.8 kg·mol^{-1}, $Đ$ = 7.8). ^{1}H NMR (600 MHz, $C_2D_2Cl_4$) δ 7.95 – 7.84 (m), 7.80 (d, J = 7.8 Hz), 7.57 (t, J = 7.7 Hz), 7.48 (t, J = 7.5 Hz), 5.83 (d, J = 9.1 Hz), 5.53 – 5.43 (m), 4.31 – 4.10 (m), 2.49 – 2.39 (m), 2.16 – 2.03 (m), 1.78 – 1.66 (m), 1.39 (br), 1.09 – 0.82 (m). ^{13}C NMR (151 MHz, $C_2D_2Cl_4$) δ 144.07, 142.60, 141.32, 141.05, 134.73, 130.44, 128.96, 127.76, 127.49, 127.43, 127.38, 125.77, 125.12, 50.27, 37.79, 35.48, 34.20, 33.82, 32.44, 31.93, 30.19, 29.71, 29.31, 29.23, 26.96, 26.58, 26.07, 23.14, 22.64, 14.03.

4. *Synthesis of small-molecule models*

N-cyclohexylbenzamide[1] and *N*-octylbenzamide[1] were prepared according to previously reported procedures.

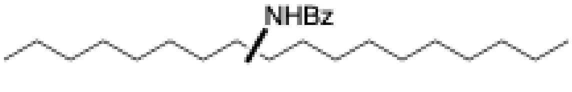

BzHN–$C_{18}H_{37}$

BzHN–$C_{18}H_{37}$: The title compound was prepared as a mixture of isomers to resemble the distribution of isomers in the functionalized polyethylenes. The title compound was prepared according to the representative protocol with octadecane (500 mg, 17.7 mmol C_2H_4 units, 1 equiv), 1,2-DCB (1 mL),

(phen)$CuCl_2$ (5.5 mg, 0.017 mmol, 0.1 mol%), benzamide (86.5 mg, 0.714 mmol, 4 mol%), and $^tBuOO^tBu$ (264 µL, 1.44 mmol, 8 mol%) for 4 h. The crude reaction mixture was poured into 5 mL MeOH and the precipitate was filtered. The resulting solid was purified by column chromatography (0% → 30% EtOAc/hexanes) to yield a beige wax (143 mg, 19% yield relative to octadecane). The ^1H NMR spectrum of the title compound (mixture of isomers) is shown below (Figure S27).

5. *Synthesis of amides*

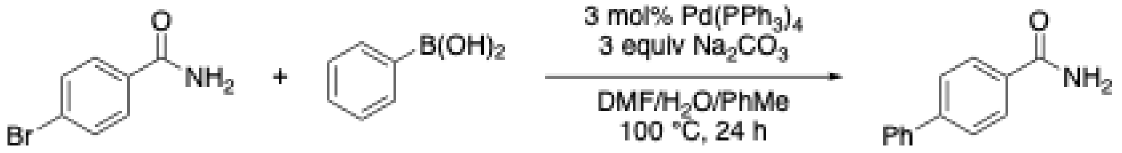

4-phenyl-benzamide: To a 50 mL round-bottom flask were added 2.00 g 4-bromobenzamide (1 equiv, 10.0 mmol), 1.46 phenylboronic acid (1.2 equiv, 12.0 mmol), 347 mg $Pd(PPh_3)_4$ (3 mol%, 0.3 mmol), 3.18 g Na_2CO_3 (3 equiv, 30 mmol), 5 mL DMF, 5 mL H_2O, and 5 mL PhMe. The reaction mixture was heated at 100 °C for 24 h. Then, the reaction mixture was diluted with H_2O (50 mL) and extracted with EtOAc (3x50 mL). The organic layer was washed with brine (50 mL), dried over $MgSO_4$, and concentrated under vacuum. The title compound was purified by column chromatography (5% MeOH/DCM) to yield a pale-yellow solid (1.17 g, 59% yield). The NMR spectra of the title compound match previously reported data.[11]

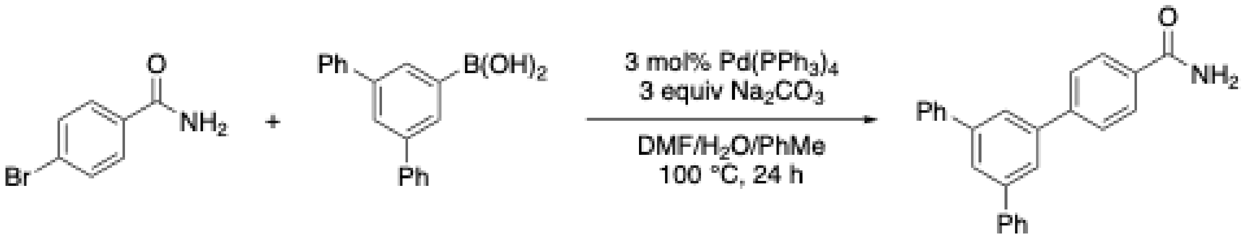

4-(3,5-diphenylphenyl)-benzamide: To a 50 mL round-bottom flask were added 2.00 g 4-bromobenzamide (1 equiv, 10.0 mmol), 3.29 g (3,5-diphenylphenyl)boronic acid (1.2 equiv, 12.0 mmol), 347 mg $Pd(PPh_3)_4$ (3 mol%, 0.3 mmol), 3.18 g Na_2CO_3 (3 equiv, 30 mmol), 5 mL DMF, 5 mL H_2O, and 5 mL PhMe. The reaction mixture was heated at 100 °C for 24 h. Then, the reaction mixture was diluted with H_2O (50 mL) and extracted with EtOAc (3x50 mL). The organic layer was washed with brine (50 mL), dried over $MgSO_4$, and concentrated under vacuum. The title compound was purified by column chromatography (30% → 100% EtOAc/hexanes) to yield a yellow solid, which was further purified by washing with cold EtOAc to yield a colorless solid (2.10 g, 60% yield). ^1H NMR (500 MHz, $CDCl_3$) δ 7.94 (app. d, J = 7.5 Hz, 2H), 7.86 – 7.74 (m, 5H), 7.70 (app. d, J = 7.4 Hz, 4H), 7.50 (app. t, J = 7.7 Hz,

4H), 7.41 (app. t, J = 7.1 Hz, 2H), 6.07 (br, 2H). ^{13}C NMR (126 MHz, $CDCl_3$) δ 169.20, 144.90, 142.75, 141.16, 141.01, 132.34, 129.06, 128.14, 127.86, 127.66, 127.49, 126.12, 125.30. HRMS (ESI-TOF) calcd. For $[C_{25}H_{20}NO]^+$ $(M+H)^+$: *m/z* 350.1545, found 350.1541.

6. Mechanical and Thermal properties

Table S1. Summary of results of tensile tests

Polymer	Tensile stress at max load (MPa)	Young's Modulus (MPa)	Tensile strain at break (%)	Toughness (MJ/m^3)	Yield stress (MPa)
**0%–NHBz-HDPE**	26 ± 1	572 ± 57	236 ± 46	45 ± 9	26 ± 1
**1%–NHBz-HDPE**	20 ± 1	398 ± 55	407 ± 99	75 ± 15	20 ± 1
**2%–NHBz-HDPE**	26 ± 1	326 ± 30	888 ± 56	170 ± 7	16 ± 2

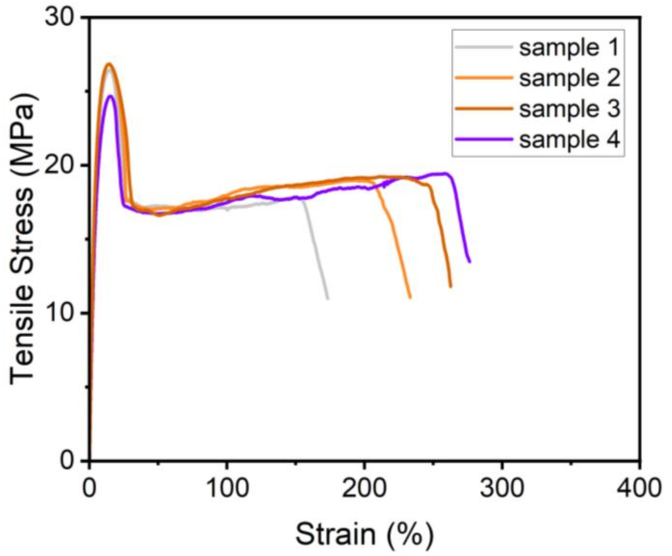

Figure S1. Tensile test curves for **0%–NHBz-HDPE** (unmodified HDPE)

Table S2. Summary of results of tensile tests for **0%–NHBz-HDPE** (unmodified HDPE)

Polymer	Tensile stress at max load (MPa)	Young's Modulus (MPa)	Tensile strain at break (%)	Toughness (MJ/m^3)	Yield stress (MPa)
Sample 1	26.45	618.56	173.15	31.87	26.45
Sample 2	26.85	620.11	233.20	45.04	26.85
Sample 3	24.67	504.78	276.32	52.01	24.67
Sample 4	26.85	546.32	262.60	49.10	26.85

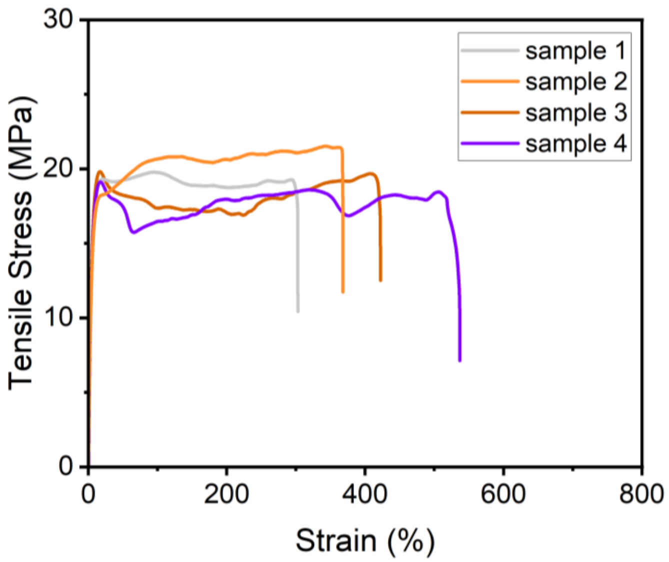

Figure S2. Tensile test curves for **1%–NHBz-HDPE**

Table S3. Summary of results of tensile tests for **1%–NHBz-HDPE**

Polymer	Tensile stress at max load (MPa)	Young's Modulus (MPa)	Tensile strain at break (%)	Toughness (MJ/m^3)	Yield stress (MPa)
Sample 1	19.81	437.19	422.09	75.90	19.81
Sample 2	19.79	324.1	302.72	57.12	19.32
Sample 3	19.12	440.67	536.70	93.91	19.12
Sample 4	21.53	388.12	367.97	74.72	20.83

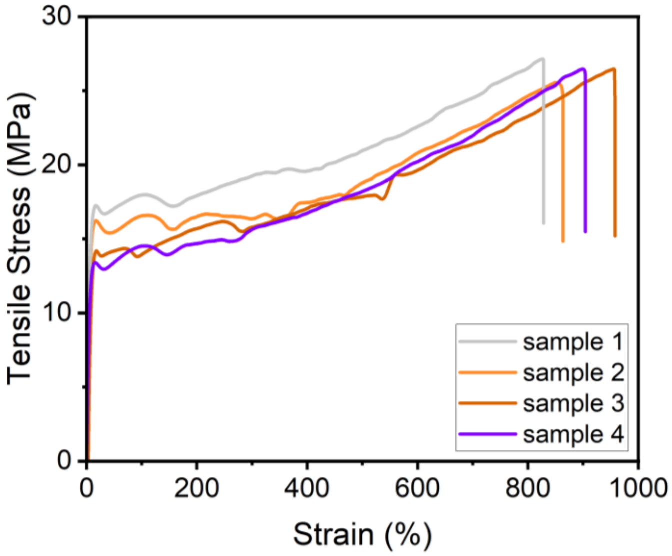

Figure S3. Tensile test curves for **2%–NHBz-HDPE**

Table S4. Summary of results of tensile tests for **2%–NHBz-HDPE**

Polymer	Tensile stress at max load (MPa)	Young's Modulus (MPa)	Tensile strain at break (%)	Toughness (MJ/m^3)	Yield stress (MPa)
Sample 1	25.57	340.26	863.42	163.62	16.6
Sample 2	26.48	302.28	957.70	178.8	14.36
Sample 3	27.15	360.69	828.23	171.12	17.99
Sample 4	26.48	300.28	903.93	166.07	14.54

Table S5. Summary of melting (T_m) temperatures, enthalpies of melting (ΔH_m) and percent crystallinity (X_c)

Polymer	T_m (°C)	ΔH_m (J/g)	X_c (%)
**0%–NHBz-HDPE**	129.3 ± 0.3	164 ± 29	56 ± 10
**1%–NHBz-HDPE**	118.5 ± 0.4	102 ± 3	35 ± 1
**2%–NHBz-HDPE**	110.5 ± 0.5	75 ± 1	26 ± 1

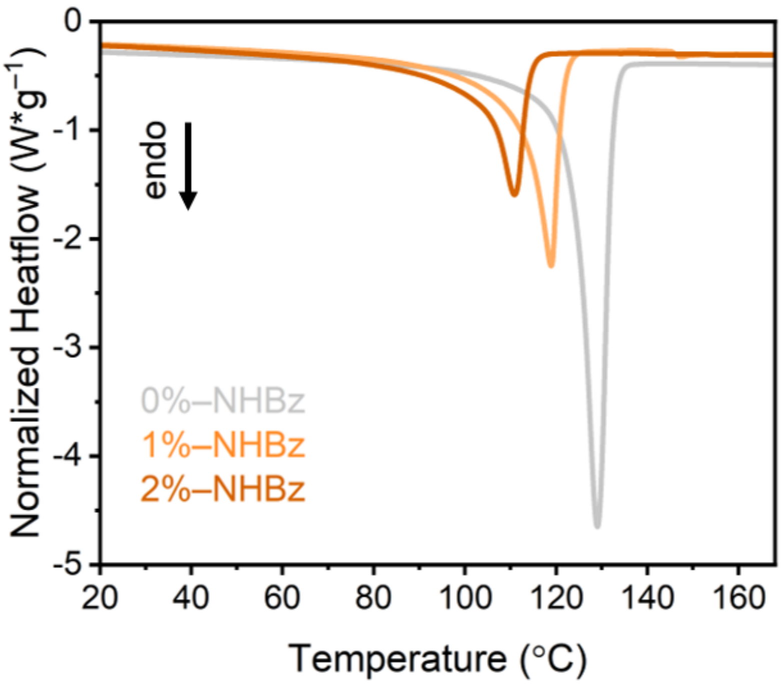

Figure S4. Representative DSC curves of HDPEs with 0, 1, and 2 mol % benzamide incorporation (gray, light orange, and orange, respectively). Data plotted from the 2nd heating cycle at a heating rate of 10 °C min^{-1}. The melt temperature and melt enthalpy of HDPE samples decreased with increasing levels of amide incorporation. From the known relationship between the melt enthalpy of the polymer and its percent crystallinity[5] we calculated the degree of crystallinity for each sample to be 66%, 35% and 26% for 0, 1, and 2 mol% HDPE respectively (see Section 1.4 for details).

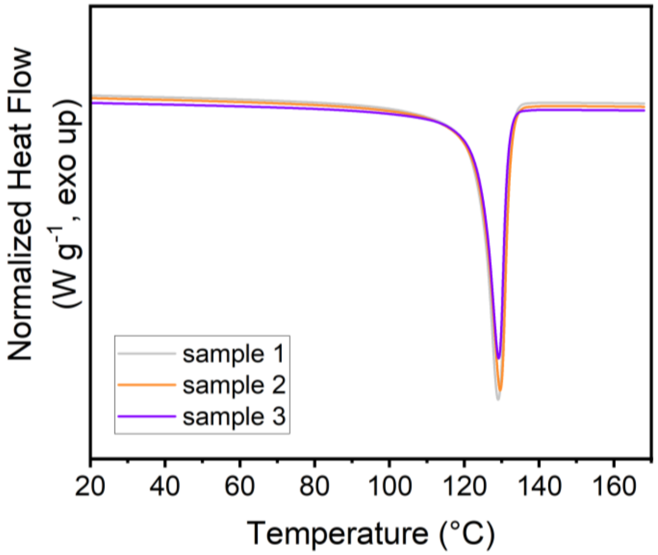

Figure S5. DSC curves of **0%–NHBz-HDPE**. T_m = 129.3 ± 0.3 °C, ΔH_m = 164 ± 29 J/g, X_c = 56 ± 10%.

Table S6. Summary of results of DSC for **0%–NHBz-HDPE**

**Polymer**	T_m **(°C)**	ΔH_m **(J/g)**	X_c (%)
Sample 1	129.02	191.98	65.5
Sample 2	129.59	165.80	56.6
Sample 3	129.17	134.06	45.8

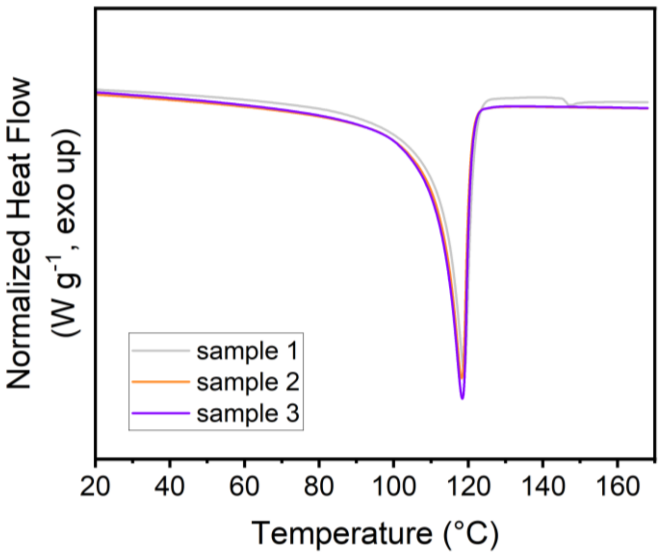

Figure S6. DSC curves of **1%–NHBz-HDPE**. T_m = 118.5 ± 0.4 °C, ΔH_m = 102 ± 3 J/g, X_c = 35 ± 1%.

Table S7. Summary of results of DSC for **1%–NHBz-HDPE**

**Polymer**	T_m **(°C)**	ΔH_m **(J/g)**	X_c (%)
Sample 1	118.91	102.48	35.0
Sample 2	118.23	98.22	33.5
Sample 3	118.39	104.65	35.7

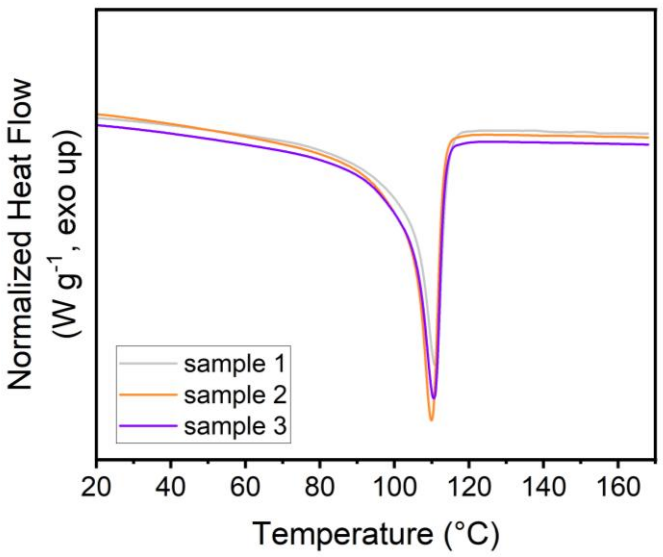

Figure S7. DSC curves of **2%–NHBz-HDPE**. T_m = 110.5 ± 0.5 °C, ΔH_m = 75 ± 1 J/g, X_c = 26 ± 1%.

Table S8. Summary of results of DSC for **2%–NHBz-HDPE**

**Polymer**	T_m **(°C)**	ΔH_m **(J/g)**	X_c (%)
Sample 1	110.94	76.115	26.0
Sample 2	110.04	74.867	25.6
Sample 3	110.66	74.164	25.3

7. Characterization of Materials

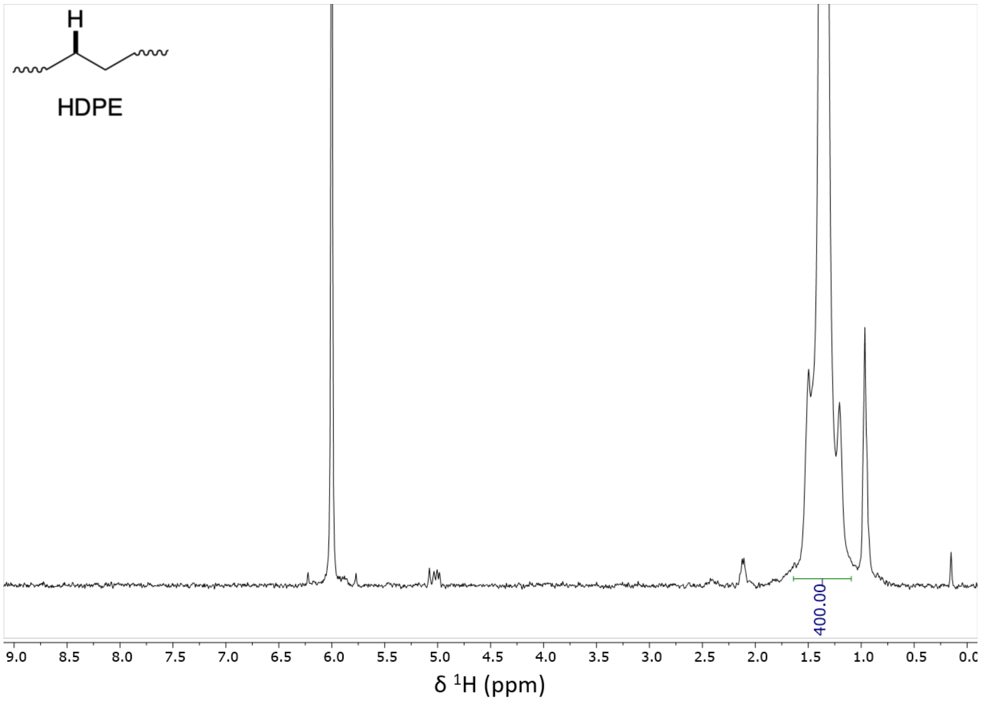

Figure S8. ^{1}H NMR spectrum of **0%–NHBz-HDPE** in $C_2D_2Cl_4$ at 100 °C. No amide groups were detected in the unmodified material.

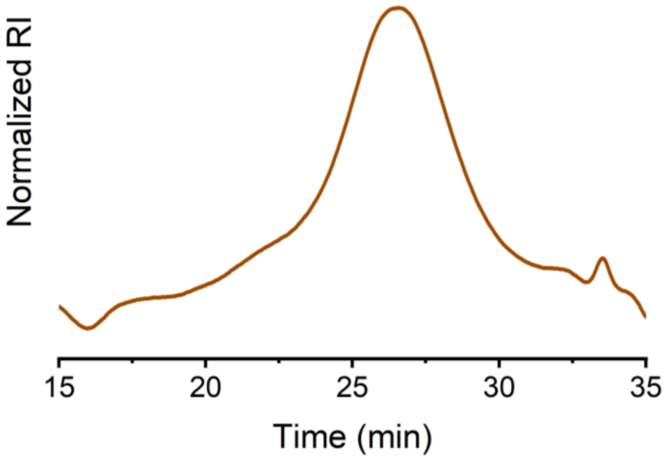

Figure S9. Gel permeation chromatogram of **0%–NHBz-HDPE**. M_n = 10.8 kg·mol^{-1}, $Đ$ = 5.0. Molecular weight was determined relative to polyethylene standards.

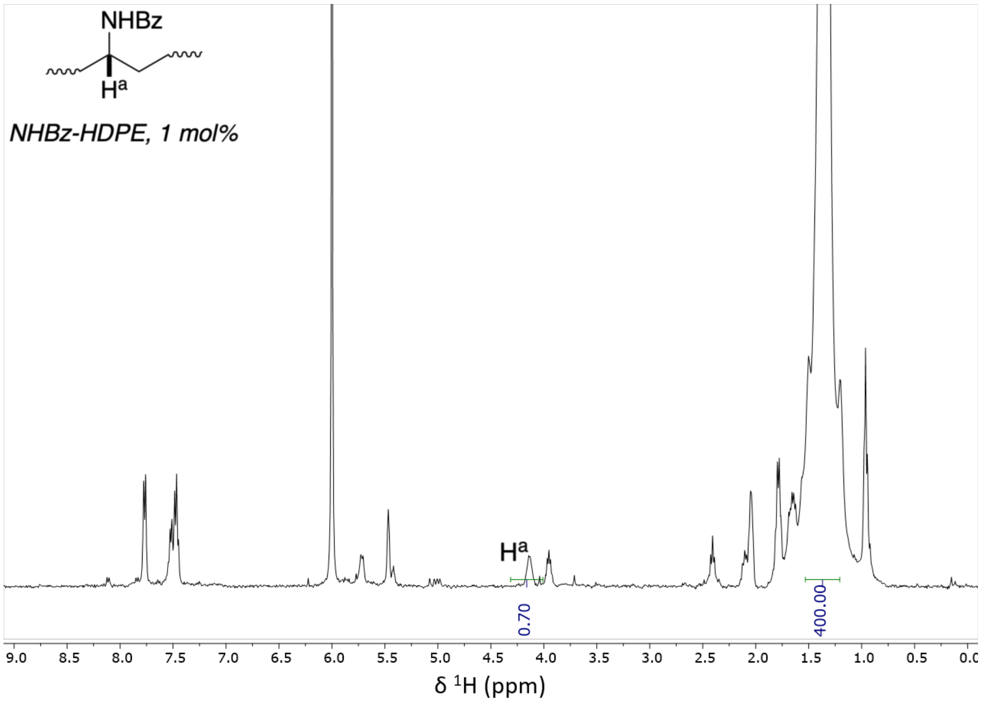

Figure S10. ^{1}H NMR spectrum of **1%–NHBz-HDPE** in $C_2D_2Cl_4$ at 100 °C. Functional group incorporation = 0.7 mol%.

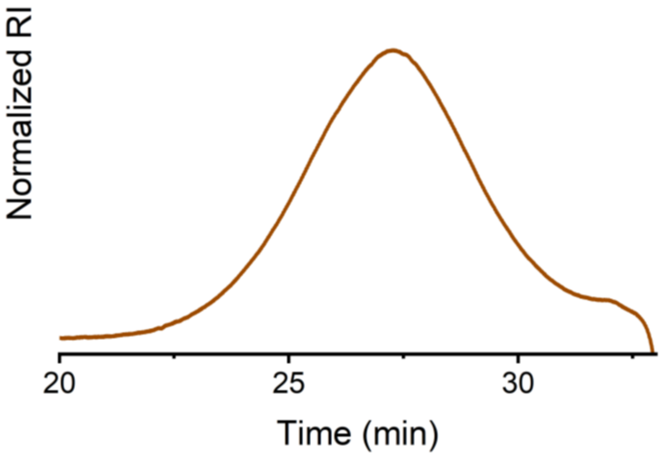

Figure S11. Gel permeation chromatogram of **1%–NHBz-HDPE**. M_n = 6.9 kg·mol^{-1}, $Đ$ = 2.8. Molecular weight was determined relative to polyethylene standards.

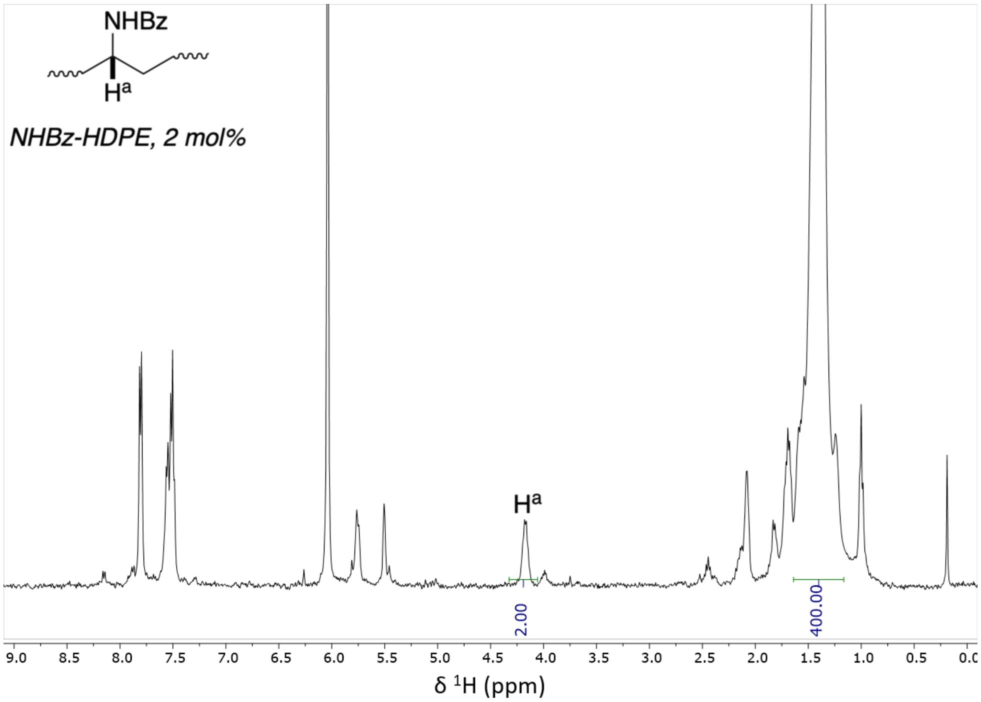

Figure S12. ^{1}H NMR spectrum of **2%–NHBz-HDPE** in $C_2D_2Cl_4$ at 100 °C. Functional group incorporation = 2.0 mol%.

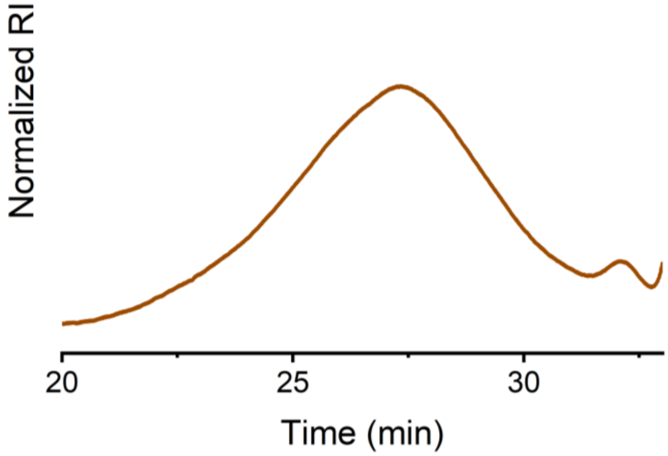

Figure S13. Gel permeation chromatogram of **2%–NHBz-HDPE**. M_n = 7.5 kg·mol^{-1}, $Đ$ = 4.0. Molecular weight was determined relative to polyethylene standards.

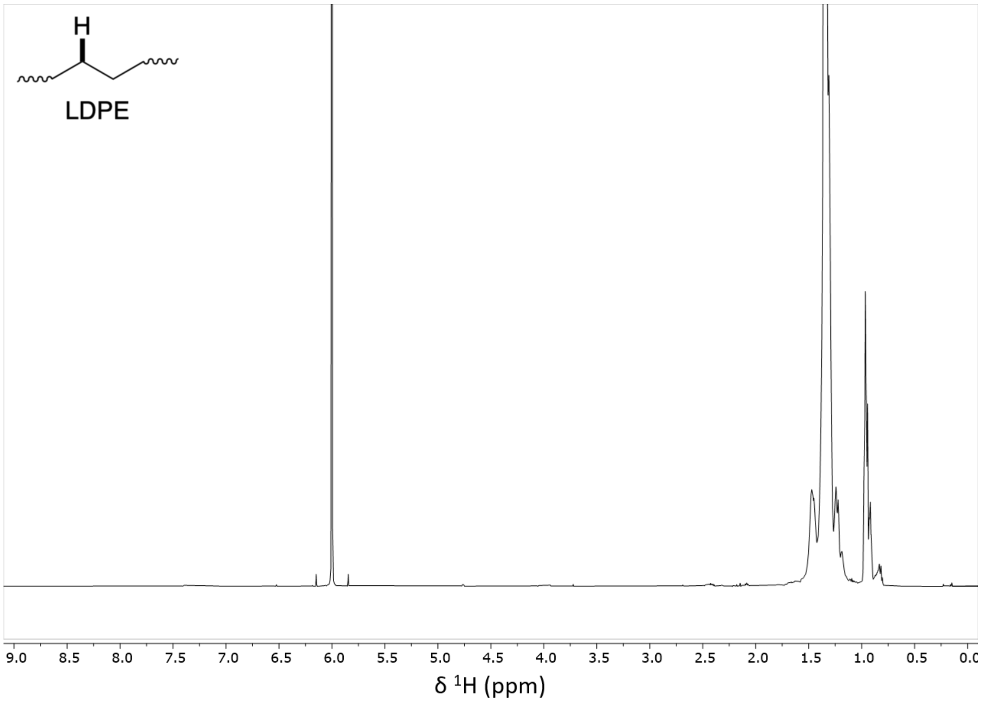

Figure S14. ¹H NMR spectrum of **0%–LDPE** in $C_2D_2Cl_4$ at 100 °C. No amide groups were detected in the unmodified material.

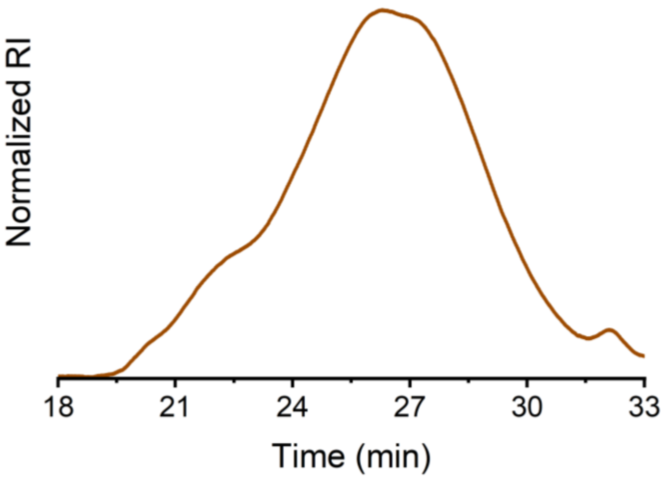

Figure S15. Gel permeation chromatogram of **0%–LDPE**. M_n = 9.5 kg·mol^{-1}, $Đ$ = 6.9. Molecular weight was determined relative to polyethylene standards.

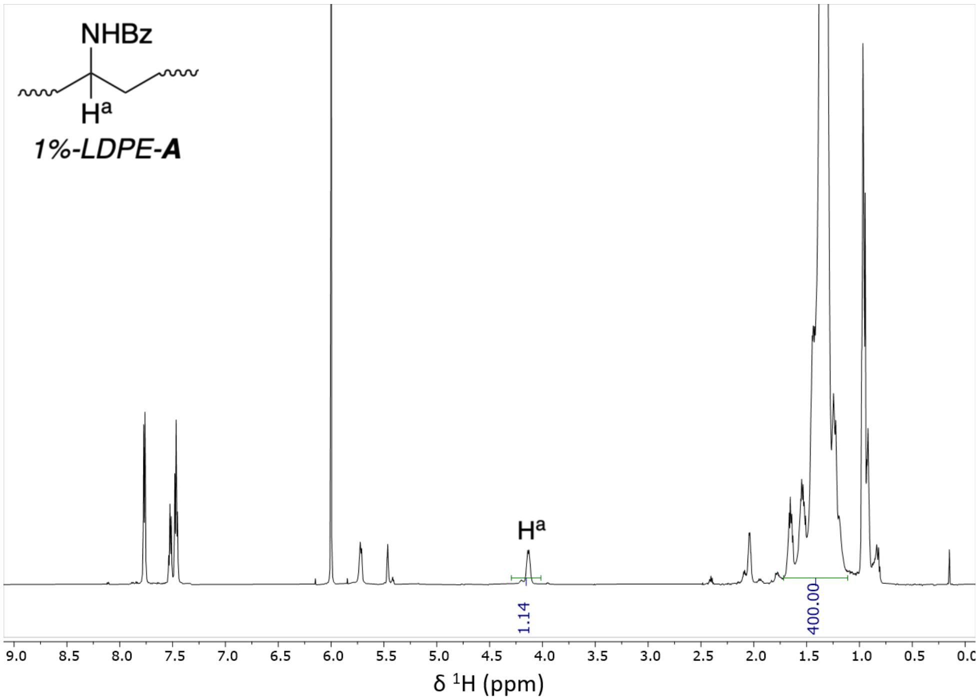

Figure S16. ^{1}H NMR spectrum of **1%–LDPE-A** in $C_2D_2Cl_4$ at 100 °C. Functional group incorporation = 1.1 mol%.

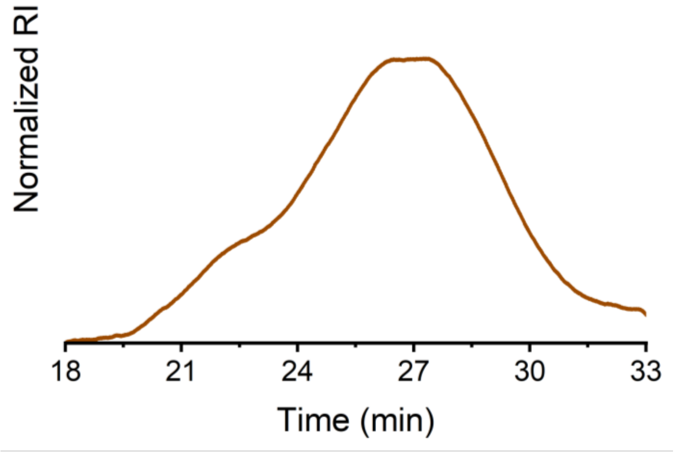

Figure S17. Gel permeation chromatogram of **1%–LDPE-A**. M_n = 7.2 kg·mol^{-1}, $Đ$ = 8.1. Molecular weight was determined relative to polyethylene standards.

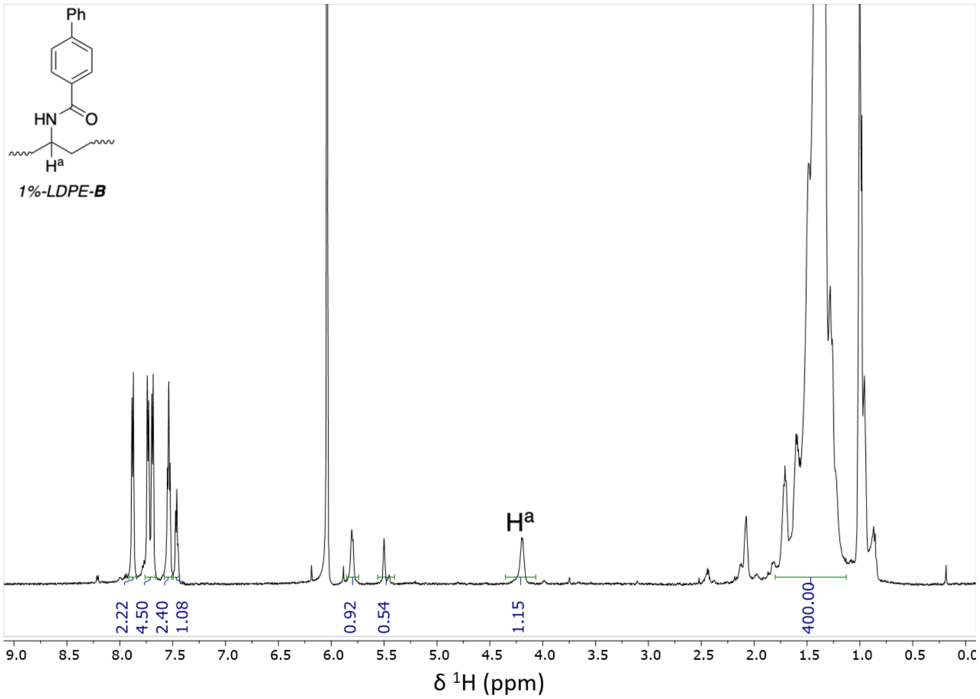

Figure S18. ^{1}H NMR spectrum of **1%–LDPE-B** in $C_2D_2Cl_4$ at 100 °C. Functional group incorporation = 1.2 mol%.

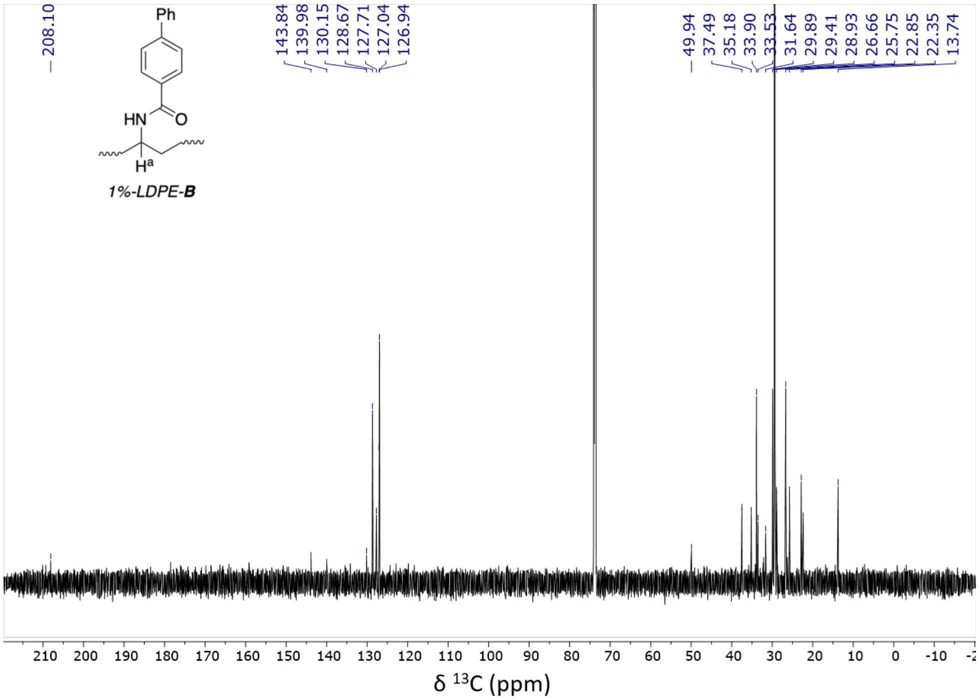

Figure S19. ^{13}C NMR spectrum of **1%–LDPE-B** in $C_2D_2Cl_4$ at 100 °C.

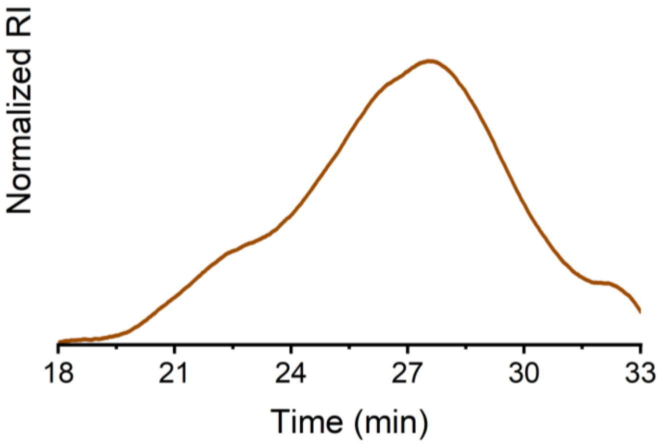

Figure S20. Gel permeation chromatogram of **1%–LDPE-B**. M_n = 6.7 kg·mol^{-1}, $Đ$ = 8.2. Molecular weight was determined relative to polyethylene standards.

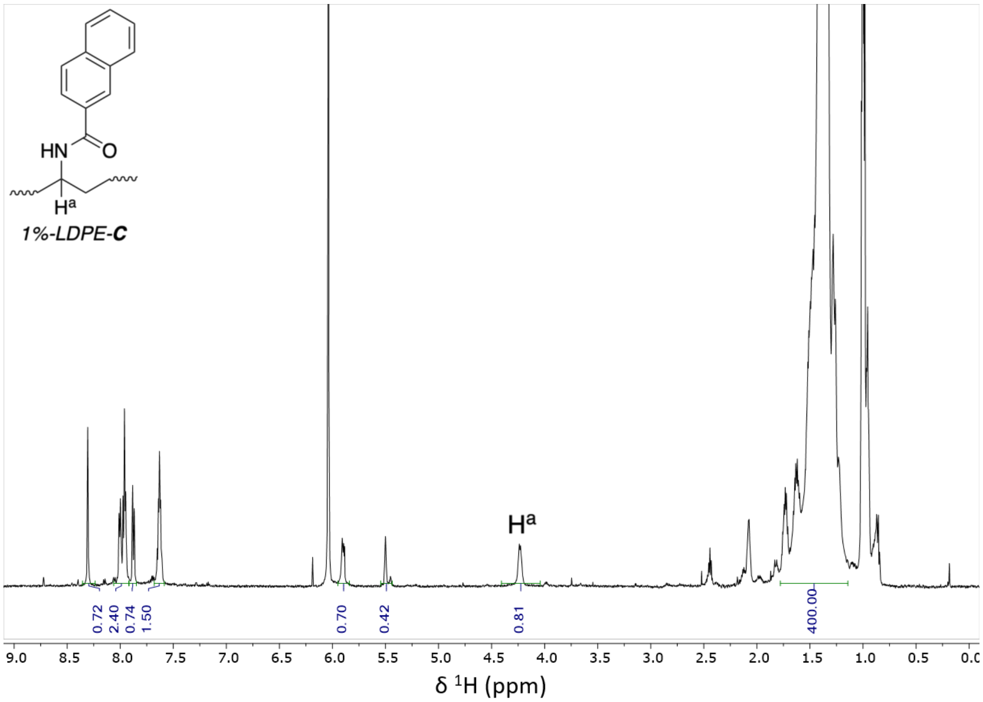

Figure S21. ^{1}H NMR spectrum of **1%–LDPE-C** in $C_2D_2Cl_4$ at 100 °C. Functional group incorporation = 0.8 mol%.

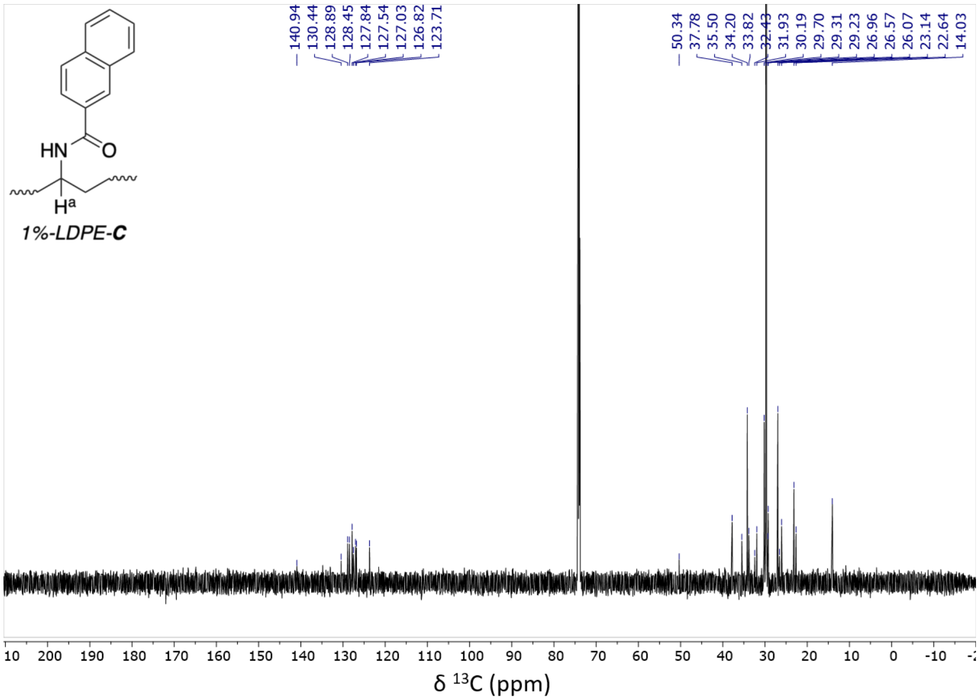

Figure S22. ^{13}C NMR spectrum of **1%–LDPE-C** in $C_2D_2Cl_4$ at 100 °C.

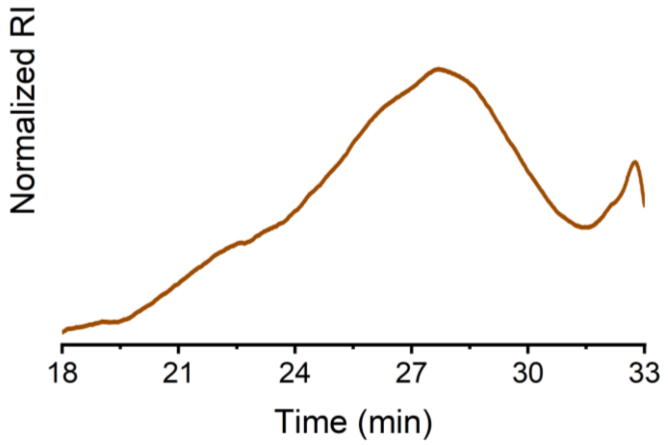

Figure S23. Gel permeation chromatogram of **1%–LDPE-C**. M_n = 7.0 kg·mol^{-1}, $Đ$ = 7.6. Molecular weight was determined relative to polyethylene standards.

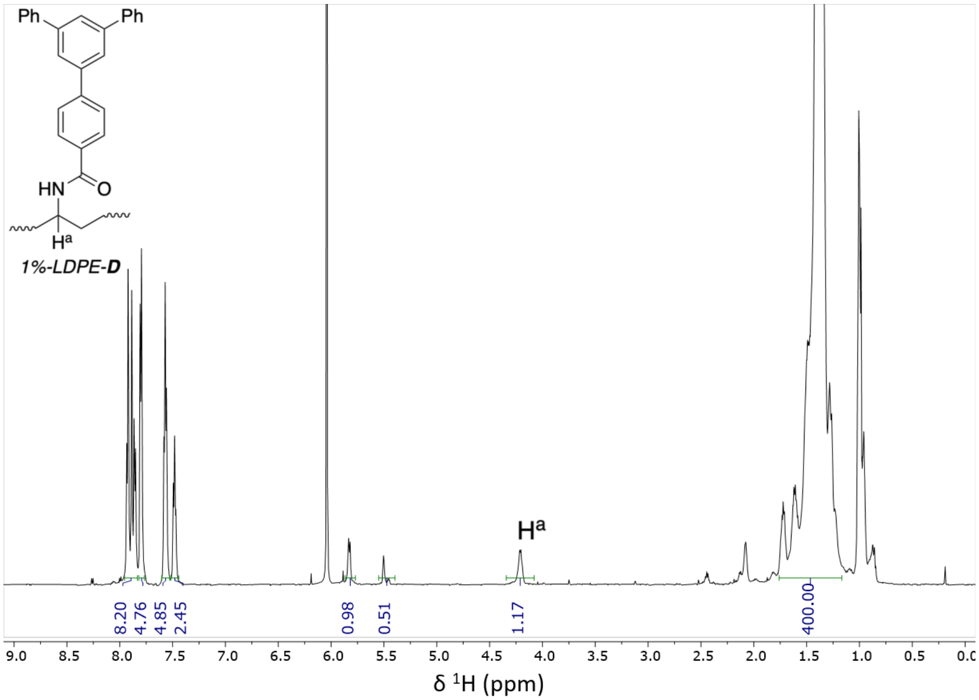

Figure S24. ^{1}H NMR spectrum of **1%–LDPE-D** in $C_2D_2Cl_4$ at 100 °C. Functional group incorporation = 1.2 mol%.

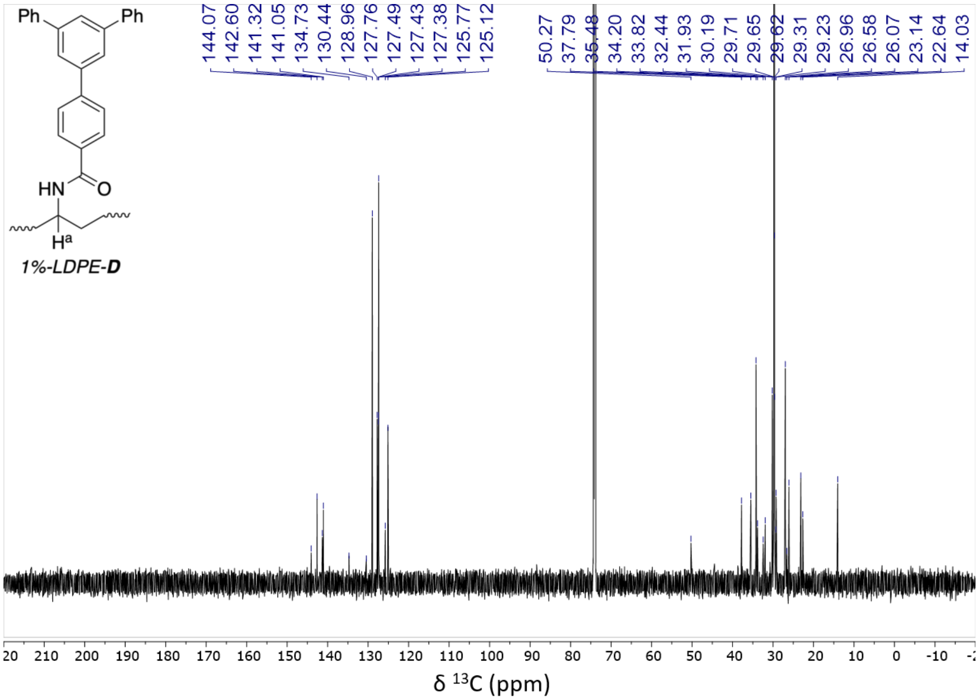

Figure S25. ^{13}C NMR spectrum of **1%–LDPE-D** in $C_2D_2Cl_4$ at 100 °C.

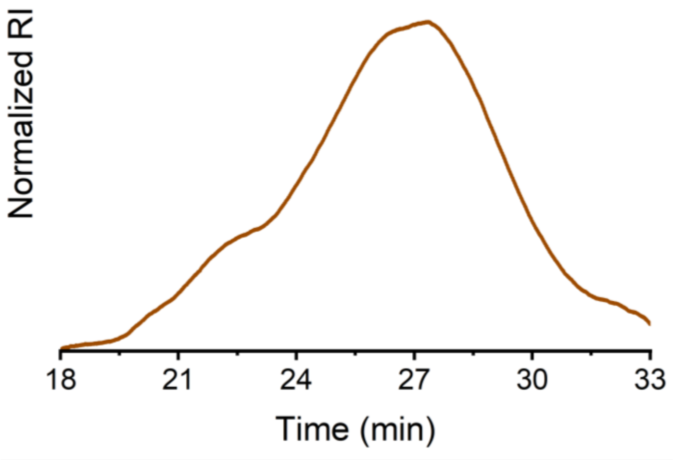

Figure S26. Gel permeation chromatogram of **1%–LDPE-D**. M_n = 7.8 kg·mol^{-1}, $Đ$ = 7.8. Molecular weight was determined relative to polyethylene standards.

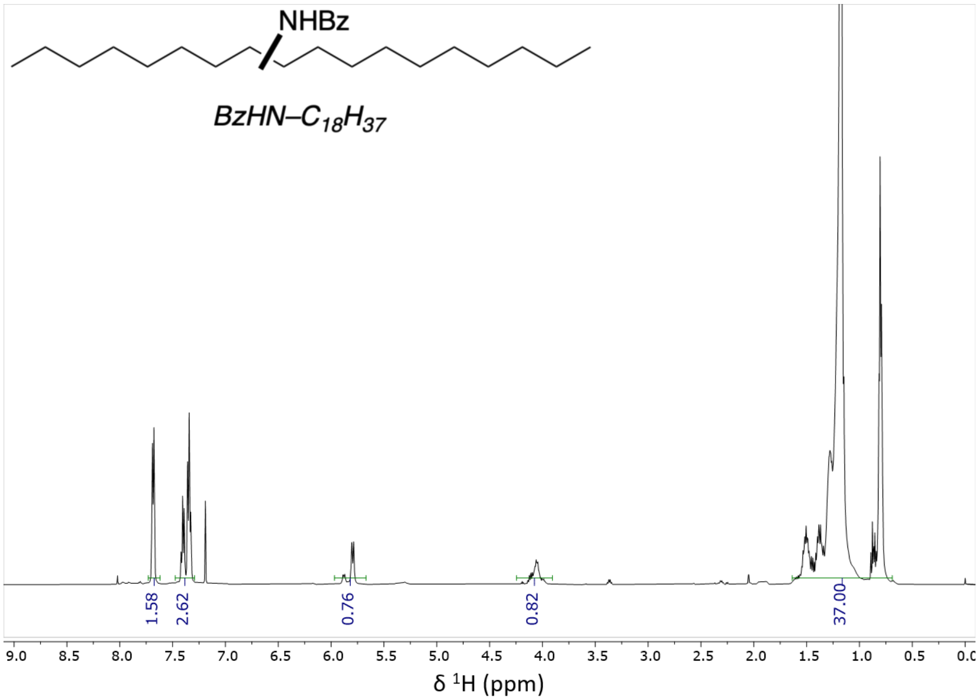

Figure S27. ^{1}H NMR spectrum of **BzHN–$C_{18}H_{37}$** in $CDCl_3$. The title compound was prepared as a mixture of isomers.

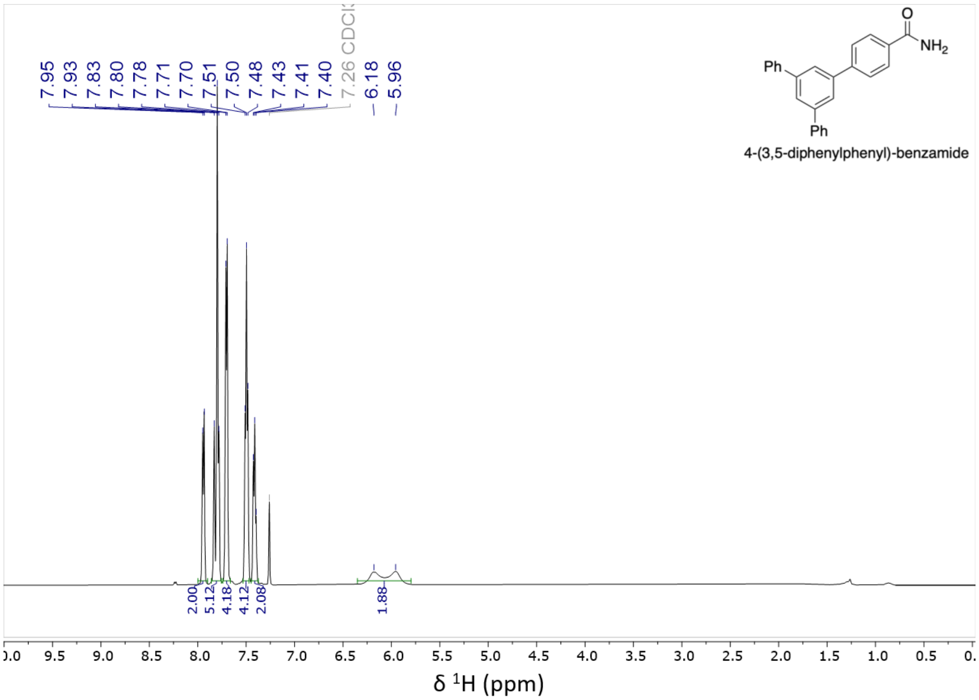

Figure S28. ^{1}H NMR spectrum of **4-(3,5-diphenylphenyl)-benzamide** in $CDCl_3$.

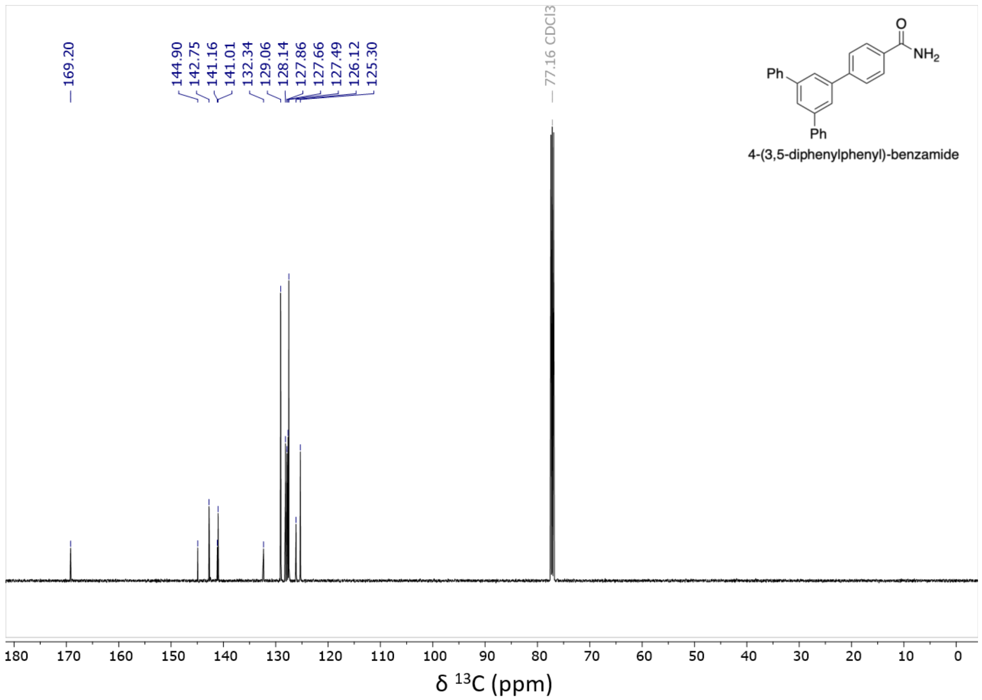

Figure S29. ^{13}C NMR spectrum of **4-(3,5-diphenylphenyl)-benzamide** in $CDCl_3$.

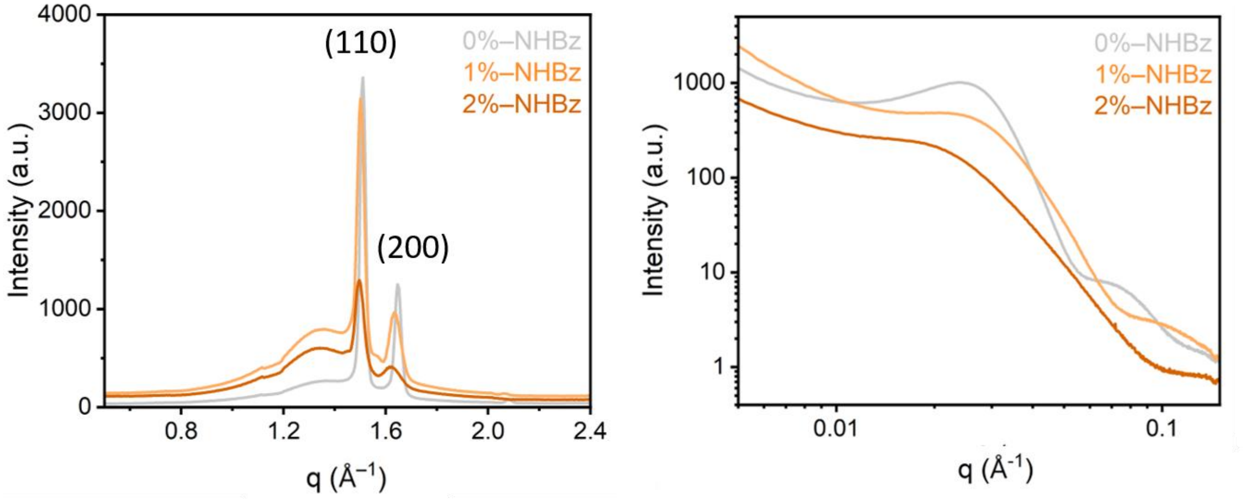

Figure S30. WAXS (left) and SAXS (right) measurements of HDPEs with 0, 1, and 2 mol % benzamide incorporation. Unit cell peaks (110) and (200) are labeled. As shown, the orthorhombic unit cell of polyethylene is observed in the WAXS measurements with apparent (110) and (200) peaks (corresponding to q values of 1.51 Å^{-1} and 1.65 Å^{-1} for unmodified HDPE, respectively).[12,13] For the polyethylenes containing amide groups, the intensity of both (110) and (200) peaks was lower than that of unmodified HDPE. A notable change in the linewidth and a shift to lower q values for both peaks was observed in these samples (see Table S10 for nominal q values). Given that the sum of the area of (110) and (200) peaks, relative to the overall sum of amorphous halo peak located at ~1.4 Å^{-1} and crystalline peaks corresponds to the percent crystallinity of the material (Experimental section, section 1.6), this value was calculated for each sample and found to be 53%, 28%, and 20% for 0, 1, and 2 mol% HDPE respectively (Table S11).

From the SAXS data: the percent of crystallinity was calculated from the thickness (d_c) of the lamella stack (crystalline phase) and that (d_a) of the amorphous phase based on the linear stacked model (vide infra and Experimental section 1.6),[14] resulting in values of 40%, 36%, and 31% for 0, 1, and 2 mol% HDPE, respectively. Analysis by both X-ray scattering techniques revealed a decrease in crystallinity with increasing amide incorporation.

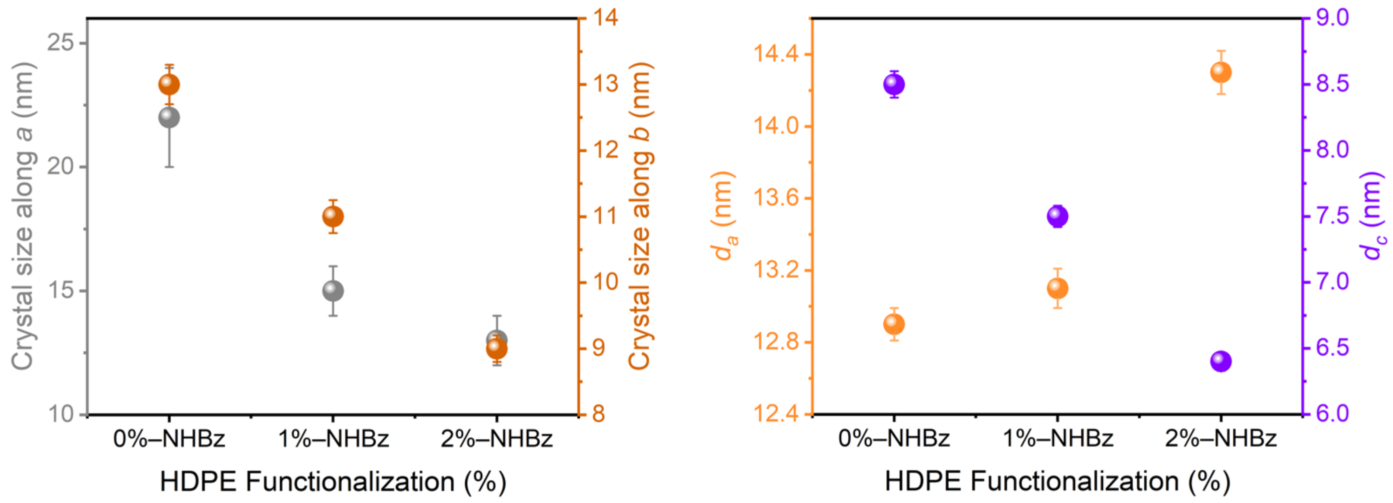

Figure S31. Crystal size (left) along the orthorhombic unit cell a direction (gray) and along b direction (orange) for HDPEs with 0, 1, and 2 mol % benzamide incorporation, determined by WAXS. Mean thickness (right) of the amorphous phase, d_a (orange), and mean thickness of the crystalline phase, d_c (purple), of HDPEs with 0, 1, and 2 mol % benzamide incorporation, determined by SAXS.

In both *a* and *b* directions, a broadening in linewidth was observed with increasing percent functionalization, suggesting that the crystal size along both directions in the lateral direction decreases with this increasing functionalization. In addition, we calculated the mean thickness of both the crystalline phase and the amorphous phase of the amide-containing HDPEs, based on the data from SAXS measurements. These calculations were based on a linear stack model; the morphology of the semicrystalline polyethylenes was assumed to be a lamellar two-phase system consisting of crystalline and amorphous phases with different electron densities.[14]

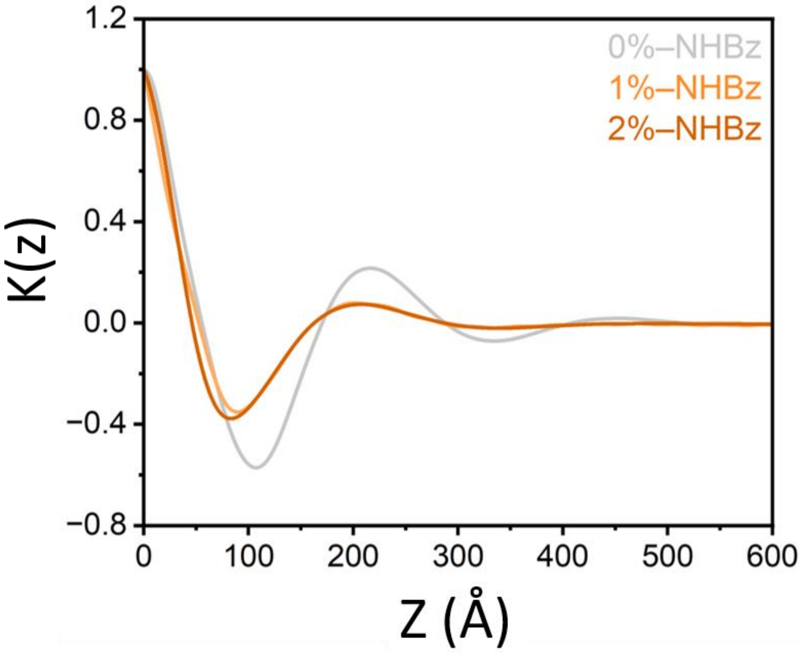

Figure S32. Correlation function K(z) obtained from SAXS data (see Figure S30) for HDPEs with 0, 1, and 2 mol % benzamide incorporation. From the correlation function, we determined the mean thickness of the crystalline phase to be 8.5 nm, 7.5 nm and 6.4 nm and the amorphous phase to be 12.9 nm, 13.1 nm and 14.3 nm for 0, 1, and 2 mol% HDPE, respectively.

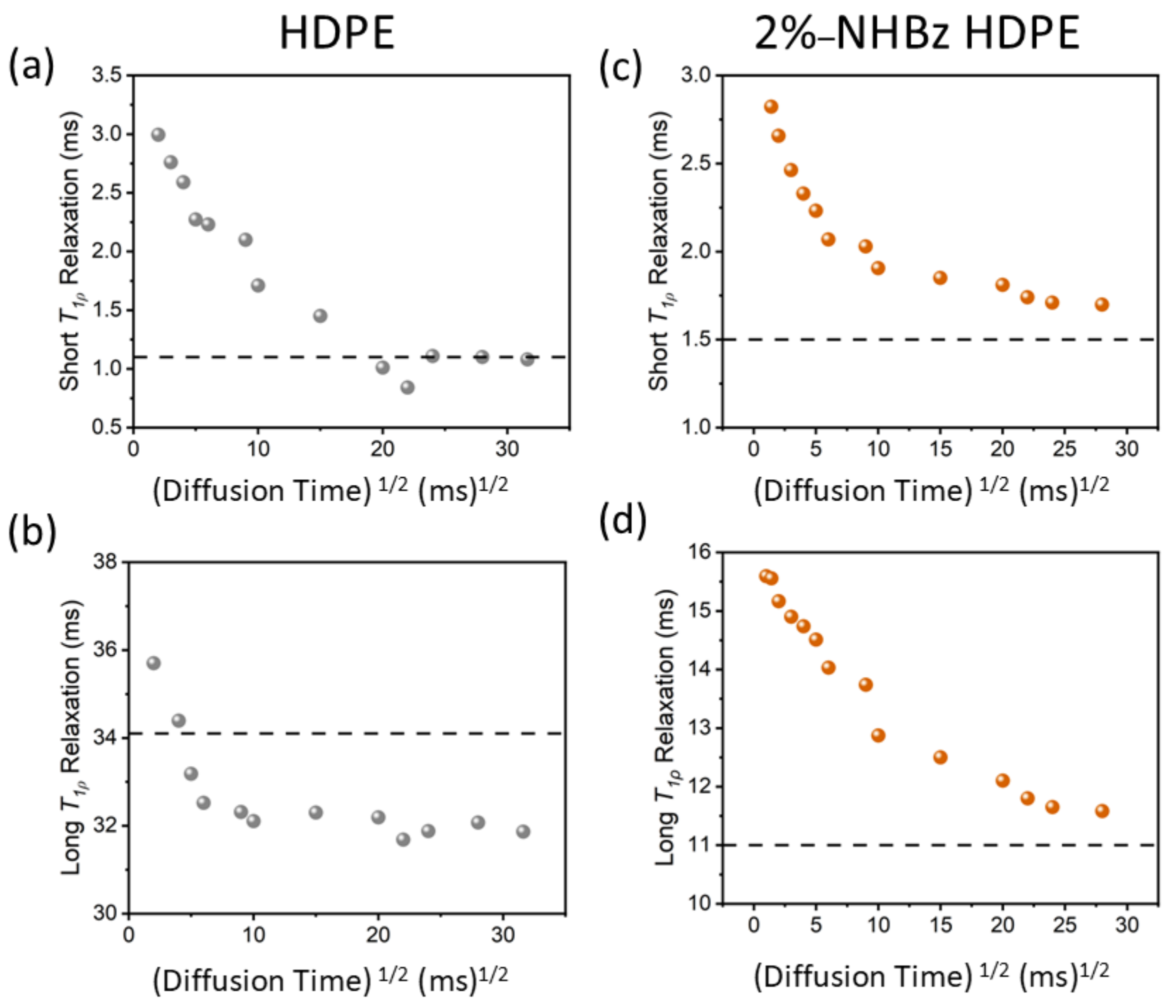

Figure S33. (a), (c) Short and (b), (d) long $T_{1\rho}$ as a function of the square root of the diffusion time (τ_d) for HDPE and 2%–NHBz HDPE, respectively. Dashed lines indicate $T_{1\rho}$ values obtained from a spin-lock relaxation experiment at the same spin-lock field strength (150 kHz).

The short component of $T_{1\rho}$ is apparent at very short spin diffusion times (< 2 ms), and this short component indicates that spin-diffusion occurred during the preparation stage of the experiment. Furthermore, the $T_{1\rho}$ values of 2%–NHBz HDPE at short spin-diffusion times are large, specifically for the long $T_{1\rho}$ component (> 15 ms). Prior work suggests that this observation could results from the presence of a boundary layer (i.e. RAF) that inhibits molecular motion in the amorphous phase.[2] This time dependency also could result from inhomogeneity of the polymer sample (such as defects and structural disorder or anisotropy). At longer spin-diffusion times, the time dependency is negligible.

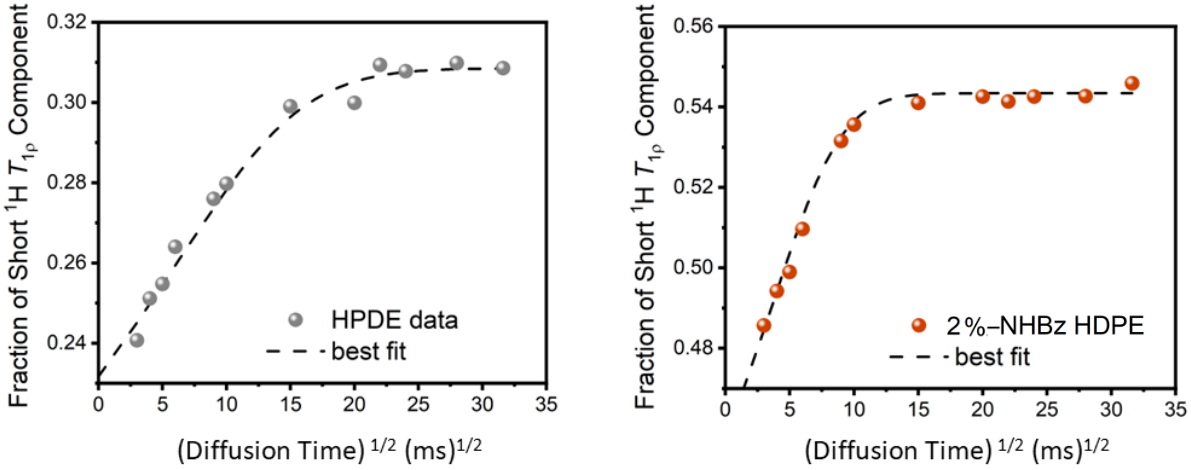

Figure S34. Short $T_{1\rho}$ component fraction as a function of the square root of the mixing time, $\sqrt{\tau_D}$, for HDPE and 2% HDPE. The filled circles are the data points and the dashed line shows the best fit from the numerical simulation. Crystallite width for HDPE and 2%–NHBz HDPE was found to be 24 and 11 nm, respectively.

The size of the crystallites can be calculated according to the correlation between spin diffusion and distance by using the following mean square distance equation: $< L^2 > = 4D\tau/3$[15] in which L is the calculated domain size, D is the diffusion coefficient found to be $5 \cdot 10^{-12}$ cm^2 s^{-1},[2] and τ is the diffusion time. Additional modified ^{1}H $T_{1\rho}$ measurements were conducted (shown in figure above) that excluded the signal from short $T_{1\rho}$ components and, thus, ensured that the presence of a peak from amorphous regimes originated from spin diffusion from the crystalline chains (details of the experimental method can be found in the experimental section 1.2). The crystallite size, according to proton spin diffusion, was found to be 24 and 11 nm for the unmodified HDPE and the 2%–NHBz HDPE, respectively, and this trend agrees with the average crystal size calculated from the SAXS measurement in Figure S31.

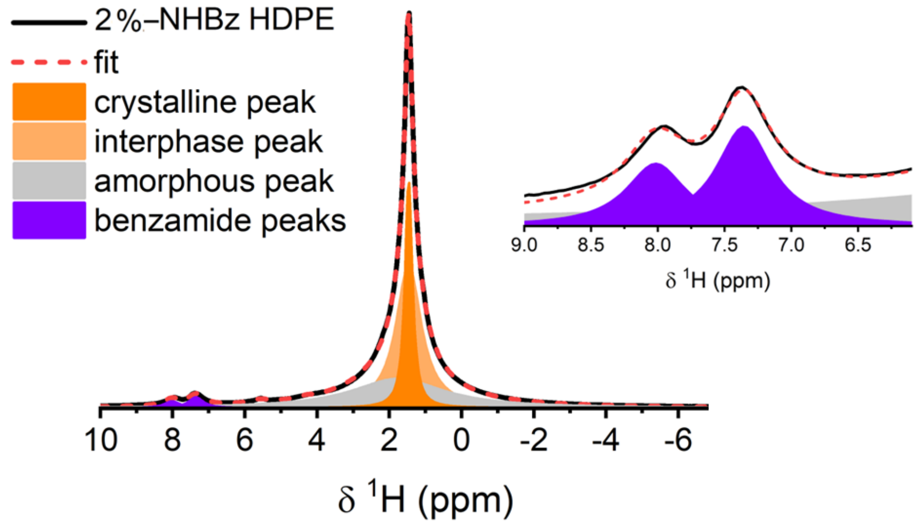

Figure S35. Representative ^{1}H $T_{1\rho}$ spectrum of 2%–NHBz HDPE, deconvoluted into crystalline, interphasial, amorphous peaks[16] and benzamidyl peaks. On the right is a zoomed figure of the benzamidyl peaks. Deconvolution of each peak was performed using DMfit software[4] 2–3 times to ensure repeatability, with the error estimation of 10–15% (as shown in Figure 3b–c). See Figure S36 for discussion of the quality of fits at varying spin-lock delays.

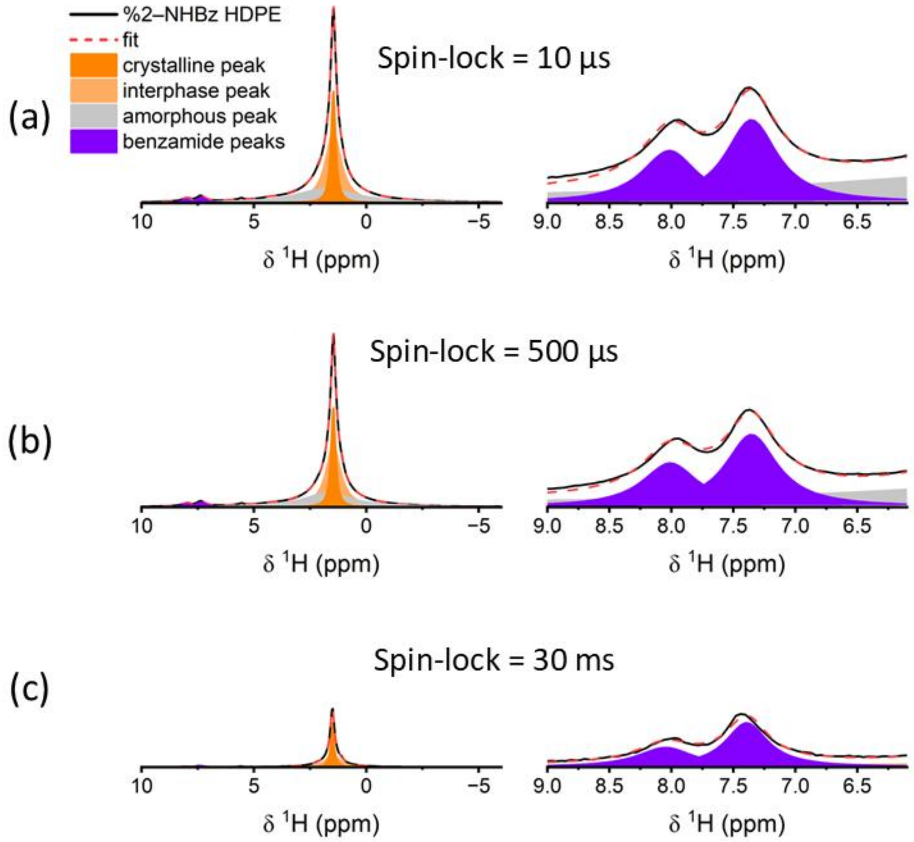

Figure S36. Representative ^{1}H $T_{1\rho}$ spectra of 2%–NHBz HDPE, deconvoluted into crystalline, interphasial, amorphous[16] and benazamidyl peaks at spin-lock delay of (a) 10 µs, (b) 500 µs, and (c) 30 ms performed with DMfit software.[4] Zoomed figures of the benzamidyl peaks are shown to the right of each spectrum. These figures show the sensitivity of NMR to small peaks in the presence of large peaks and illustrate that the fit for the small benzamidyl peaks is accurate even in the presence of the large methylene peaks.

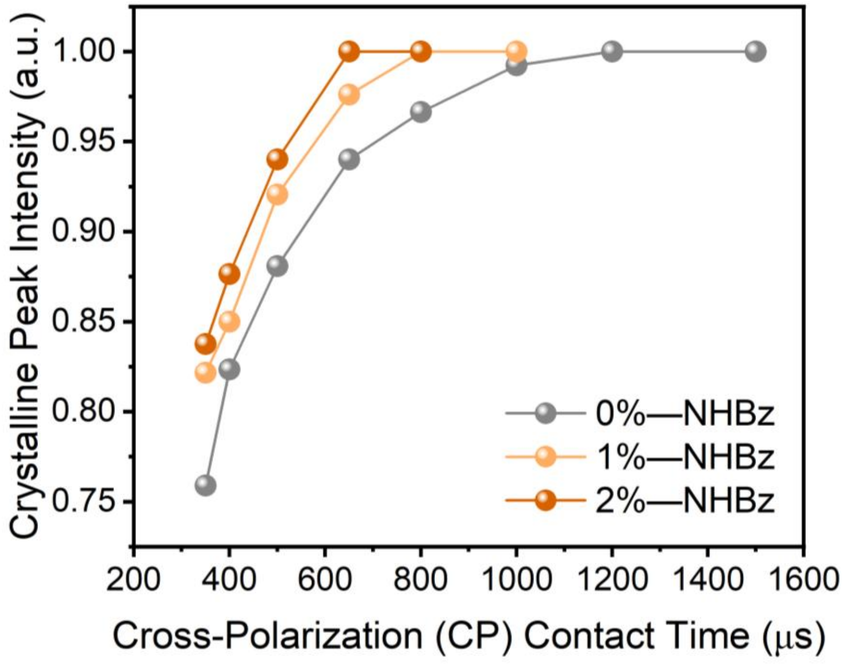

Figure S37. Signal intensity of the crystalline peak vs the ^{1}H-^{13}C CP contact time for 0%, 1%, and 2% functionalized HDPE.

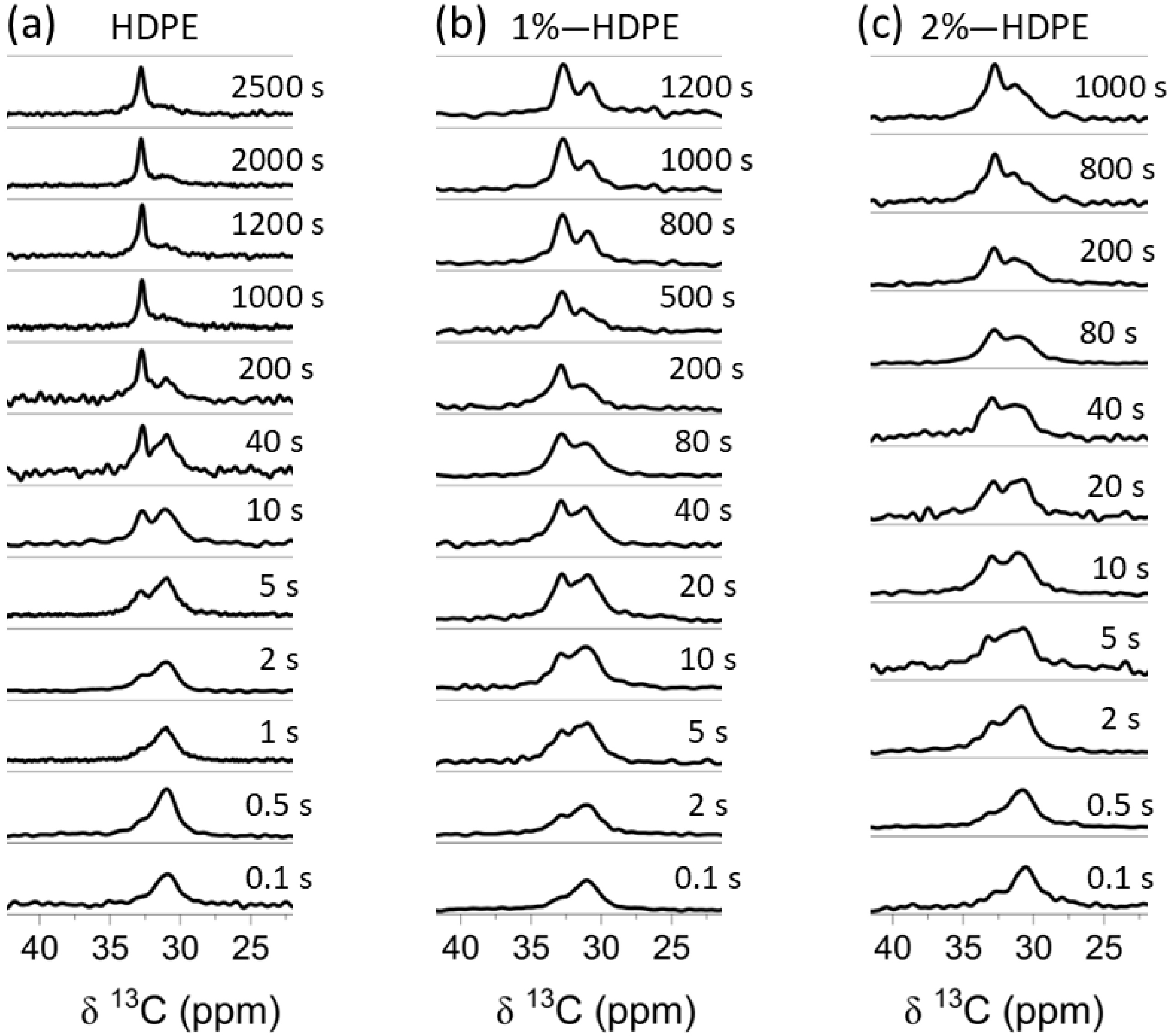

Figure S38. ^{13}C direct polarization measurements of (a) unmodified HDPE (b) 1 mol% and (c) 2m mol% benzamide incorporation, with increasing recovery delay values. The crystalline peak, resonating at ~ 33 ppm, was integrated and plotted in Figure 5c. Spectra were acquired at 11.75 T.

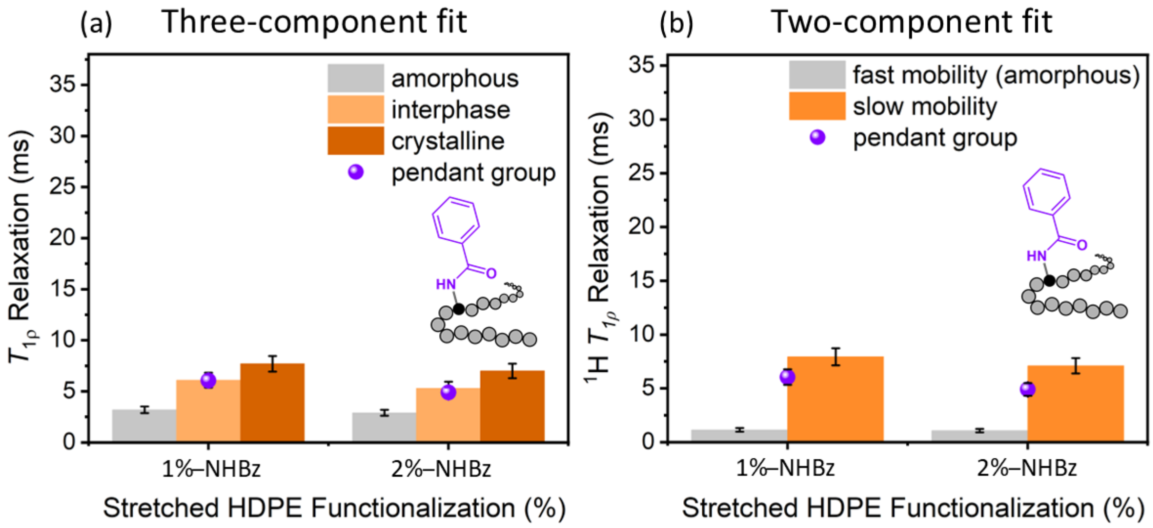

Figure S39. ^{1}H $T_{1\rho}$ relaxation rates of stretched functionalized HDPEs (1–2 mol% NHBz) recovered after tensile testing derived from a (a) three-component model and a (b) two-component model. The gray represents the fastest motion (amorphous phase), the light orange represents the interphasial (RAF) phase in the three-component model, the dark orange represents the slowest motion (crystalline phase in the three-component model), and the purple represents the pendant group (benzamide).

In the three-component model, the differences between the RAF mobility and the crystalline mobility are not large. Moreover, it is not clear whether the polymer retains its crystalline phase after stretching. Thus, we performed a two-component fit for which two distinct mobilities of the polymer are apparent. From this model, it is clear that the mobility of the pendant groups is most similar to that of the slow-moving chains.

Table S9. Summary of percentages from the three-component exponential fit for ^{1}H $T_{1\rho}$

	Amorphous	Interphase	Crystalline
**Unmodified HDPE**	17%	27%	56%
**1%-NHBz-HDPE**	18%	46%	36%
**2%-NHBz-HDPE**	20%	52%	28%

Table S10. Nominal q values from WAXS measurement of the polymer materials.

	(110) [Å $^{-1}$]	(200) [Å $^{-1}$]
**Unmodified HDPE**	1.51	1.65
**1%-NHBz-HDPE**	1.50	1.64
**2%-NHBz-HDPE**	1.498	1.63

Table S11. Summary of % of crystallinity from different methods.

	ssNMR	WAXS	SAXS	DSC
**Unmodified HDPE**	56%	53%	40%	66%
**1%-NHBz-HDPE**	36%	28%	36%	35%
**2%-NHBz-HDPE**	28%	20%	31%	26%

Table S12. Nominal ^{13}C T_1 relaxation values for the unmodified and functionalized HDPE.

	**Non–crystalline peak [s]**	**Crystalline peak [s]**
**Unmodified HDPE**	0.28	1016
**1%-NHBz-HDPE**	0.26	487
**2%-NHBz-HDPE**	0.21	412

Table S13. Nominal ^{1}H $T_{1\rho}$ relaxation values for the small-molecule model compounds.

	**^{1}H $T_{1\rho}$ [ms]**
***N*-cyclohexylbenzamide**	15
***N*-octylbenzamide**	31
***N*-$C_{18}H_{37}$ -benzamide**	12

References

[1] N. R. Ciccia, J. X. Shi, S. Pal, M. Hua, K. G. Malollari, C. Lizandara-Pueyo, E. Risto, M. Ernst, B. A. Helms, P. B. Messersmith, J. F. Hartwig, "Diverse functional polyethylenes by catalytic amination" *Science* 2023, **381**, 1433–1440.

[2] K. R. Amundson, J. A. Reimer, M. M. Denn, "Investigation of Microstructure in Poly[(p-hydroxybenzoic acid)-co-(ethylene terephthalate)] Using Nuclear Magnetic Resonance Spectroscopy" *Macromolecules* 1991, **24**, 3250–3260.

[3] B. M. Fung, A. K. Khitrin, K. Ermolaev, "An Improved Broadband Decoupling Sequence for Liquid Crystals and Solids" *J. Magn. Reson.* 2000, **142**, 97–101.

[4] D. Massiot, F. Fayon, M. Capron, I. King, S. Le Calvé, B. Alonso, J. O. Durand, B. Bujoli, Z. Gan, G. Hoatson, "Modelling one- and two-dimensional solid-state NMR spectra" *Magn. Reson. Chem.* 2002, **40**, 70–76.

[5] J. Cheng, M. Fone, V. N. Reddy, K. B. Schwartz, H. P. Fisher, B. Wunderlich, "Identification and quantitative analysis of the intermediate phase in a linear high-density polyethylene" *J Polym Sci B Polym Phys* 1994, **32**, 2683–2693.

[6] J. Ilavsky, "Nika: software for two-dimensional data reduction" *J. Appl. Cryst.* 2012, **45**, 324–328.

[7] E. K. Neidhart, M. Hua, Z. Peng, L. T. Kearney, V. Bhat, F. Vashahi, E. J. Alexanian, S. S. Sheiko, C. Wang, B. A. Helms, F. A. Leibfarth, "C-H Functionalization of Polyolefins to Access Reprocessable Polyolefin Thermosets" *J Am Chem Soc* 2023, **145**, 27450–27458.

[8] C. G. Vonk, A. P. Pijpers, "X-ray Diffraction Study of Nonlinear Polyethylene: I. Room-Temperature Observations." *Journal of Polymer Science. Part A-2, Polymer Physics* 1984, **23**, 2517–2537.

[9] D.-M. Smilgies, "Scherrer grain-size analysis adapted to grazing-incidence scattering with area detectors" *J Appl Crystallogr* 2009, **42**, 1030–1034.

[10] Z. Wang, M. Schaller, A. I. Petzold, K. I. Saalwächter, T. Thurn-Albrecht, "How entanglements determine the morphology of semicrystalline polymers" *PNAS* 2023, **120**, e2217363120.

[11] C. Tang, N. Jiao, "Copper-catalyzed aerobic oxidative C-C bond cleavage for C-N bond formation: From ketones to amides" *Angew. Chem. Int. Ed.* 2014, **53**, 6528–6532.

[12] A. Keller, "A note on single crystals in polymers: Evidence for a folded chain configuration" *Philosophical Magazine* 1957, **2**, 1171–1175.

[13] C. W. Bunn, "The Crystal Structure of Long-Chain Normal Paraffin Hydrocarbons. The 'Shape' of the CH2 Group" *Transactions of the Faraday Society* 1939, **35**, 482–491.

[14] A. Seidlitz, T. Thurn-Albrecht in *Polymer Morphology: Principles, Characterization, and Processing* (Ed.: Q. Guo), John Wiley & Sons Inc., **2016**, pp. 153–164.

[15] J. R. Havens, D. L. VanderHart, “Morphology of Poly(ethylene terephthalate) Fibers as Studied by Multiple-Pulse 1H NMR” *Macromolecules* 1985, **18**, 1663–1676.

[16] C. Hedesiu, D. E. Demco, R. Kleppinger, A. A. Buda, B. Blümich, K. Remerie, V. M. Litvinov, “The effect of temperature and annealing on the phase composition, molecular mobility and the thickness of domains in high-density polyethylene” *Polymer* 2007, **48**, 763–777.
